# Ultra‐Fast Isothermal Formation of DNA Nanostructures in Culture Media: Application to In Situ Assembly of DNA Origami With Living Cells

**DOI:** 10.1002/smll.202509401

**Published:** 2025-12-23

**Authors:** Laura Bourdon, Gerrit David Wilkens, Samy Dehissi, Salammbô Hotte, Sergii Rudiuk, Mathieu Morel, Ayako Yamada, Gaëtan Bellot, Damien Baigl

**Affiliations:** ^1^ CPCV Department of Chemistry École Normale Supérieure PSL University Sorbonne Université CNRS Paris France; ^2^ Centre de Biologie Structurale Université Montpellier CNRS Inserm Montpellier France

**Keywords:** bio‐interface, brain organoid, DNA nanotechnology, nanomachine, self‐assembly

## Abstract

Synthetic DNA strands are programmable and biocompatible building blocks that can be combined through hybridization to form user‐defined nanostructures, but their assembly traditionally requires cell‐incompatible conditions, imposing a lengthy ex situ fabrication step before any application with living matter. Here we demonstrate for the first time that 2D and 3D DNA origami structures can isothermally self‐assemble at 37°C within minutes, directly in cell culture media, both in the absence and in the presence of living cells. Scaffold‐free structures of extended dimensions, such as micrometer‐long DNA nanotubes, can also self‐assemble when the system is given more time to evolve. With human cell lines, 2D and 3D origami structures in situ self‐assemble in 5 to 15 min, and remain stable for about 24 h and up to 3 days when actin monomers are added. Similar self‐assembly performance is observed in the presence of more complex tissue‐like systems, such as human induced pluripotent stem cells evolving into cerebral organoids. This ultra‐fast, life‐compatible self‐assembly method drastically simplifies the fabrication of complex DNA nanostructures and enables the creation of in situ self‐assembling nanomachines for direct and adaptive interactions with living cells.

## Graphical abstract

We demonstrate for the first time that DNA nanostructures self‐assemble in situ at 37°C within minutes, directly in cell culture media and in the presence of living cells. The approach enables the formation of 2D and 3D DNA origami structures with human cell lines and induced pluripotent stem cells evolving into cerebral organoids.

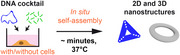

## 1 Introduction

In situ hybridization of synthetic nucleic acids within living cell environments has proved to be a powerful strategy to selectively detect and/or interact with biological targets [1], with applications ranging from biosensing [2, 3, 4] to therapeutics [5, 6, 7]. In a very different context, this ability of nucleic acids to self‐assemble through complementary base pairing has been exploited by researchers to build elaborate nanostructures in a user‐friendly and highly programmable manner, leading to the development of structural DNA nanotechnology [8, 9, 10, 11]. Within the different methods, DNA origami has emerged as one of the most widely studied and applied technologies [12, 13, 14]. Based on the guided folding of a circular single‐stranded DNA scaffold through the addition of a designed cocktail of hundreds of short oligonucleotides acting as staples, DNA origami structures can be produced at a high yield, with virtually any desired 2D [12] or 3D [15, 16] morphology [13]. With a typical size around 100 nm, these nanostructures offer precise site‐specific functionalization at subnanometric resolution [17], making them powerful platforms for nanoscale organization of matter with an exceptional degree of programmability and versatility. Among various application fields, DNA origami structures have been particularly recognized as valuable tools to interact with living organisms [18], leading to emerging applications including virus recognition and capture [19, 20], mechanobiology [21, 22, 23], synthetic immunology [24, 25, 26, 27], cancer treatment and vaccine [27, 28], or drug delivery [29, 30]. However, their conventional preparation is slow, taking hours to days, and relies on a constraining thermal annealing process (heating to around 80°C followed by a slow cooling ramp) that is incompatible with living cells. Additionally, DNA origami self‐assembly typically requires magnesium concentrations ranging from 10 to 25 mm [31], far exceeding physiological levels and posing further challenges for direct applications in biological environments. As a result, and as of today, origami structures have always been first fabricated before being added to cells for further studies. There is thus a critical scientific and methodological gap: while in situ hybridization enables nucleic acids to interact with biological targets, it currently lacks the capacity for elaborate structural organization, whereas DNA origami assembly offers exquisite programmability but requires ex situ preparation under conditions unsuitable for live cells. To bridge this gap, we thus looked for a method allowing in situ assembly of synthetic nucleic acid cocktails into user‐defined DNA origami structures directly in the presence of live cells. We recently reported that DNA origami structures could be isothermally self‐assembled in a magnesium‐free, monovalent salt‐rich buffer (e.g., Tris acetate buffer supplemented with 100 or 150 mm NaCl) at room temperature [32], but the assembly remained long (typically 24 h) and the buffer conditions were not compatible with cell culture and manipulation. In this work, our strategy was thus to look for existing cell culture buffers containing a sufficiently high amount of monovalent salts (typically around 150 mm), along with essential divalent cations (e.g., Mg^2+^, Ca^2+^) required for cell culture, but at a concentration low enough to ensure isothermal assembly conditions at a temperature favorable to live cell growth. In fact, several commercially available and widely used buffers satisfy these conditions. We discovered that directly mixing the DNA origami scaffold and staples in such buffers resulted in the isothermal self‐assembly of well‐formed DNA origami structures at 37°C in a few minutes only. This is not only one to two orders of magnitude faster than existing methods, whether they require thermal annealing or not, but also constitutes the first origami assembly conditions fully compatible with live cell maintenance, culture, and growth. We thus applied and characterized this method for the in situ programmable isothermal self‐assembly of various 2D and 3D origami morphologies directly in the presence of live cells, ranging from conventional 2D culture to more complex 3D tissue‐like systems such as cerebral organoids.

## 2 Results and Discussion

### 2.1 Ultra‐Fast DNA Origami Self‐Assembly in Cell Culture Media at 37°C

**FIGURE 1:**
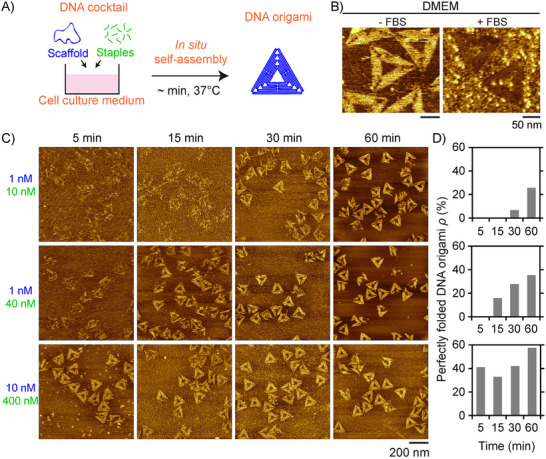
DNA origami structures self‐assemble at 37°C in only a few minutes in the widely used Dulbecco's modified Eagle's medium (DMEM) cell‐culture medium. (A) The experiment consists of the direct mixing of a DNA origami cocktail (circular single‐stranded DNA scaffold + staple mix) in a proper cell culture medium, and letting the DNA origami structures self‐assemble isothermally at 37°C, without any pre‐ or post‐thermal treatment. (B) Atomic force microscopy (AFM) images of triangular DNA origami structures obtained after 6 h incubation at 37°C in DMEM, without (left) and with (right) 10% fetal bovine serum (FBS). [scaffold]  =  10 nm; [staple] = 100 nm. (C) AFM observation of the self‐assembly of triangle origami structures over time. Each line corresponds to a given concentration in scaffold (top value indicated in blue) and staples (bottom value indicated in green). (D) Fraction of perfectly folded origami structures (*ρ*) among all detected objects by AFM for different incubation times at 37°C and the scaffold and staple concentrations shown in (C). The number of analyzed objects is given in Table S2.

Flawless formation of DNA origami is challenging, as hundreds of like‐charged DNA strands have to associate while ensuring the formation of around 10 000 to 20 000 hydrogen bonds through specific base complementarity. A typical approach to achieve this involves a thermal annealing process in the presence of a high concentration of stabilizing dications, typically magnesium (usually at a concentration ranging from 10 to 25 mm) [31]. Isothermal assembly at room temperature has been demonstrated as an alternative by replacing dications with a higher concentration of monovalent salts (typically 100 or 150 mm), ensuring both sufficient stability and reconfigurability for nearly flawless DNA assembly [32]. However, these conditions remain incompatible with cell culture and growth. To achieve isothermal assembly of DNA origami structures in the presence of live cells at 37°C, we first selected the widely used Dulbecco's modified Eagle's medium (DMEM) for its high concentration in monovalent cations (around 160 mm, Table S1) and millimolar amounts of dications (mainly Ca^2+^ and Mg^2+^, Table S1). We simply mixed a DNA origami cocktail (scaffold and staples) in DMEM and incubated it at 37°C for in situ self‐assembly (Figure 1A). With a set of staples coding for triangle origami, atomic force microscopy (AFM) revealed successful origami formation after a few hours of incubation (Figure 1B left; Figure S1 top). The experiment was repeated in the presence of 10 vol% of fetal bovine serum (FBS), a commonly required supplement for cell culture. Although the presence of high protein amount rendered the imaging of the DNA origami structures less accurate, the distinction of well‐formed triangles (Figure 1B right; Figure S1 bottom) indicated proper origami folding in these conditions as well. Isothermal self‐assembly at 37°C also led to well‐formed DNA origami structures in i) RPMI (Roswell Park Memorial Institute medium), another commonly used cell culture medium; ii) Essential 8, a medium for stem cell culture; and iii) PBS (phosphate buffered saline), a usual buffer for biological research (Figure S2). All these buffers share a concentration of monovalent cations between 140 and 160 mm and divalent ones below 3 mm (Table S1), ensuring robust isothermal self‐assembly at 37°C. To investigate the kinetics and yield of this isothermal self‐assembly, we used AFM to analyse the morphology evolution of structures formed after adding the DNA origami cocktail into FBS‐free DMEM as a function of incubation time at 37°C (Figure 1C) and established the fraction of perfectly well folded origami structures among all detected objects, referred to as *ρ* (Figure 1D; Table S2). Using conventional concentrations of scaffold (1 nm) and staples (40 nm each), we observed the formation of ill‐shaped assemblies within 5 min, which progressively evolved into well‐formed triangles after 15 min, with *ρ* progressively increasing with time, reaching 35% after 1 h only (Figure 1C,D middle). Decreasing the staple concentration to a 10‐fold excess slowed the assembly but still resulted in successful folding (*ρ =* 25%) within 1 h (Figure 1C,D top). Strikingly, increasing the scaffold concentration to 10 nm while maintaining a 40‐fold staple excess led to the formation of a significant amount of well‐folded triangles after 5 min only (*ρ =* 41%) (Figure 1C,D bottom), which represents a considerably shorter time than the hours to days needed for known methods with [12] or without [32] thermal annealing. Notably, at these high DNA concentrations, the yield did not further evolve significantly and remained high (between 33% and 57%), showing that most of the assembly process was completed in the first minutes.

**FIGURE 2:**
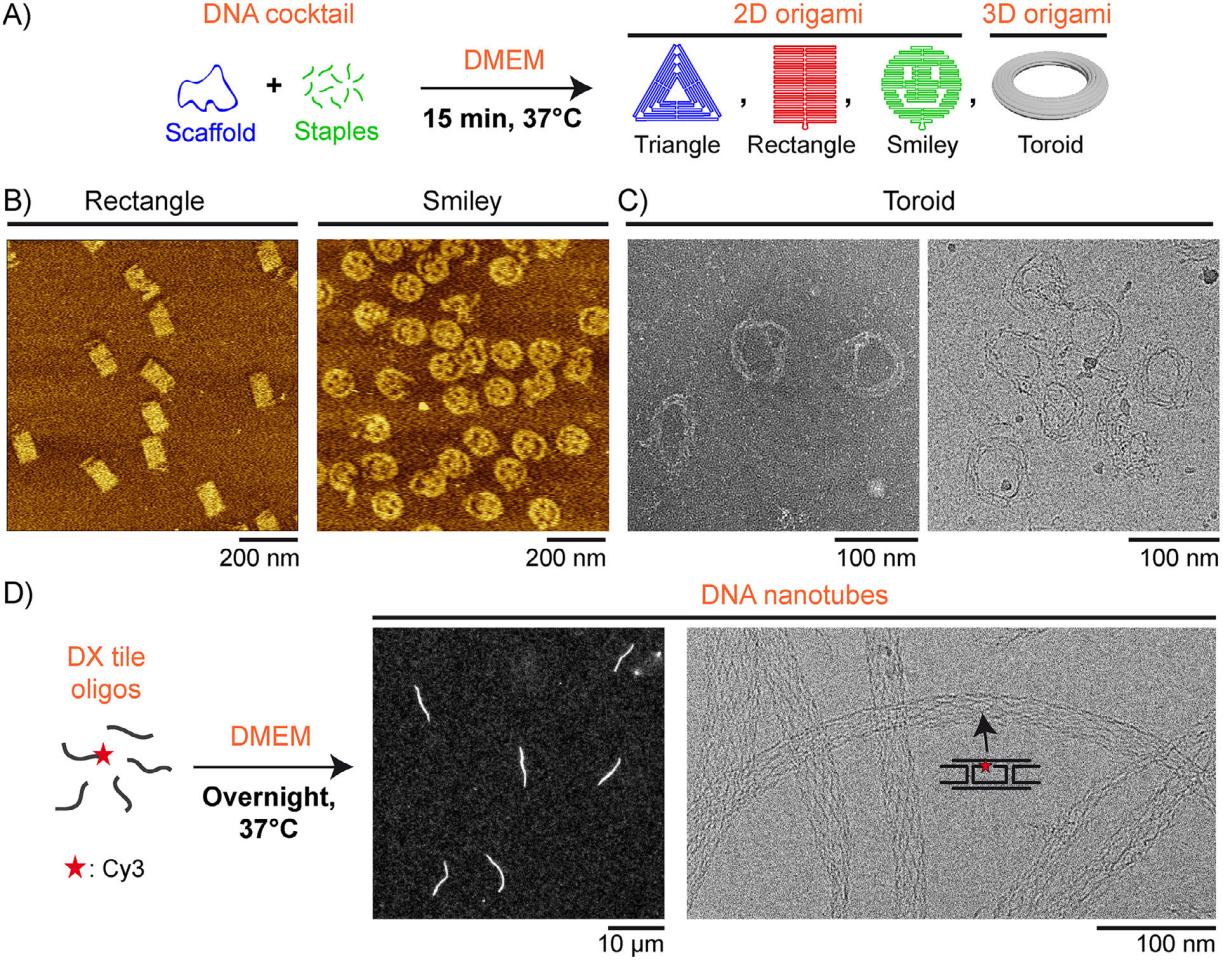
Versatile self‐assembly into user‐programmed 2D and 3D nanostructures. (A–C) DNA cocktails coding for different 2D or 3D origami structures are mixed in DMEM and left for self‐assembly at 37°C for 15 min, producing (B) rectangle or smiley shapes as observed by AFM and (C) 3D toroids as observed by transmission electron (TEM, left) and cryo‐electron (cryo‐EM, right) microscopy. [scaffold]  =  10 nm; [staple] = 400 nm (rectangle, smiley) or 100 nm (toroid). (D) 5 DNA oligonucleotides (500 nm each) forming a double‐crossover (DX) tile motif are mixed in DMEM and left for self‐assembly at 37°C overnight, leading to the formation of DNA nanotubes observed by fluorescence microscopy (left) and cryo‐EM (right). One oligonucleotide is fluorescently labeled by a Cy3 dye at the 5’ end, and a schematic of the tile is shown on the cryo‐EM image.

With the same scaffold concentration (10 nm), we assembled DNA origami cocktails coding for different morphologies in DMEM and let the self‐assembly occur at 37°C for 15 min (Figure 2A–C). Using M13mp18 scaffold and a 40‐fold excess of staples, 2D structures, including compact (rectangles) and circular (smileys) ones, formed well (Figure 2B). We also explored the challenge of a more complex design, consisting of a hollow 3D structure forming a toroid shape with a single layer of adjacent double helices. Using a p7560 scaffold and a 10‐fold excess of staples, a high number of toroidal structures were detected by AFM (Figure S3), transmission electron (TEM, Figure 2C left; Figure S4) and cryo‐electron (cryo‐EM, Figure 2C right) microscopy, with a diameter 75 ± 6 nm (mean ± SD, *n* = 25) consistent with the design (Figure S5). To evaluate the generalizability of our method to the assembly of scaffold‐free DNA nanostructures, we used a mixture of 5 oligonucleotides forming a double‐crossover (DX) tile with complementary sticky ends, which can assemble in turn to produce nanotubes. One oligonucleotide is 5’‐tagged with the Cy3 dye to allow fluorescence microscopy observations. Compared to the original design [33] previously reported to enable self‐assembly in NaCl at 25°C [34], we used longer sticky ends [35] to favor assembly at 37°C. Overnight incubation in DMEM led to individual µm‐long fluorescent filaments freely fluctuating in solution (Movie S1), which were further adsorbed on a glass cover slip for in‐plane imaging (Figure 2D, left). Cryo‐EM revealed that, although dispersed in their diameter, the filaments' inner structure consisted of precisely ordered individual DNA strands respecting the tile motif design (Figure 2D, right). All these results show that mixing all DNA strands coding for a desired nanostructure in a monovalent cation‐rich mammalian cell culture medium, characteristic of most commonly used media, results in successful isothermal self‐assembly at 37°C. Under these conditions, user‐programmed 2D and 3D DNA origami structures form within minutes, while scaffold‐free structures such as µm‐long DNA nanotubes are obtained when the system is allowed to assemble for longer times. The ultra‐fast formation of origami structures across various culture media led us to explore their in situ self‐assembly in the presence of different living cell systems, including 2D cell culture and organoid formation.

### 2.2 In Situ Origami Assembly With Live Cells in 2D Culture

**FIGURE 3:**
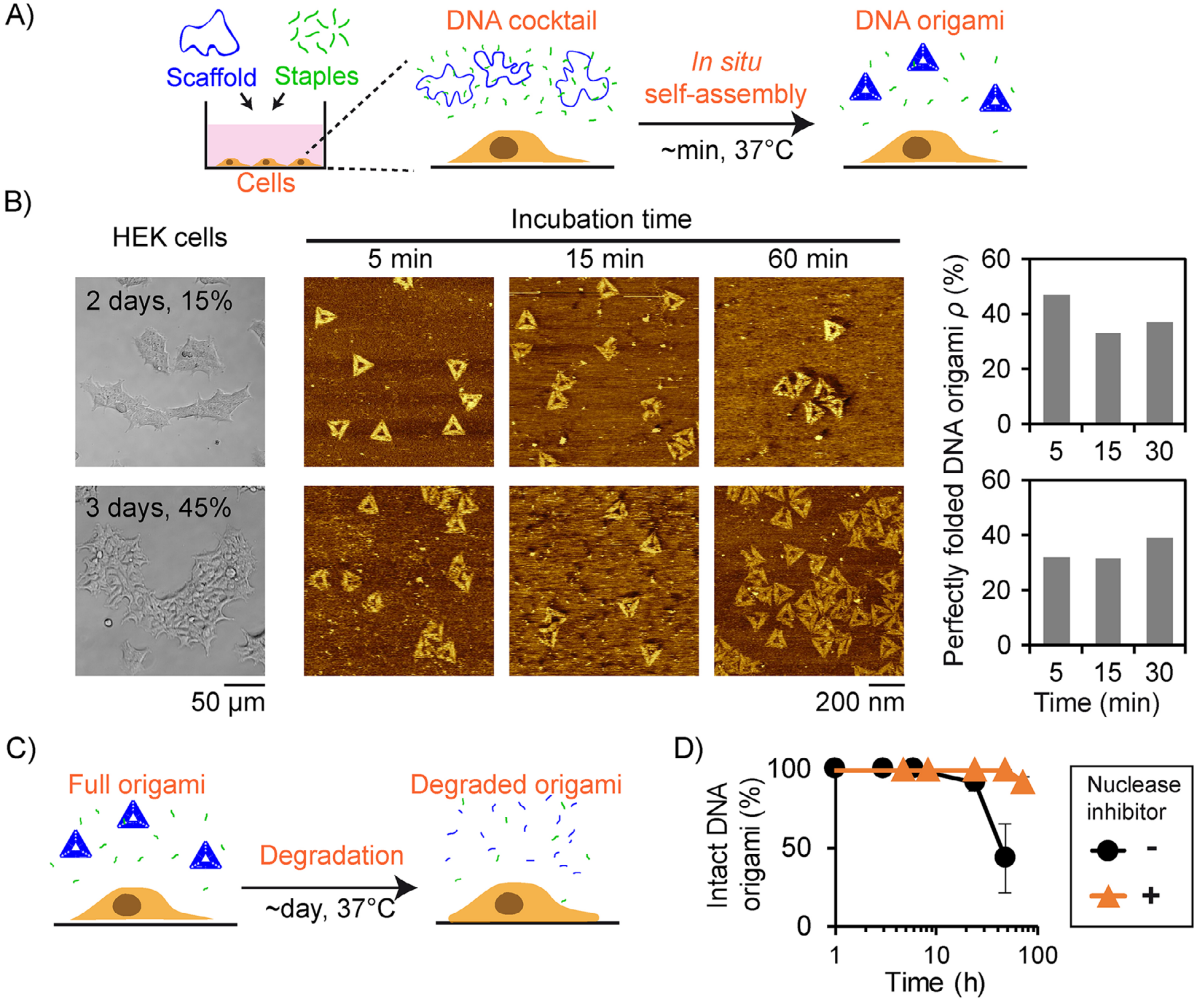
Fast self‐assembly of stable 2D origami structures in the presence of live cells (human embryonic kidney (HEK) 293T). (A) The experiment consists of adding a DNA cocktail coding for triangle origami ([scaffold] = 10 nm; [staple] = 400 nm) to cells in culture (DMEM, 10% FBS, Penicillin‐Streptomycin) and incubating the system at 37°C with 5 vol% CO_2_. (B) Transmission optical microscopy images of the cells (left), AFM images of origami structures obtained after different incubation times with cells (middle), and resulting yield of perfectly folded origami structures (right), after 2 days (15% confluence, top) or 3 days (45% confluence, bottom) of cell culture before DNA cocktail introduction. Origami samples were purified from proteins before AFM imaging using a multi‐step procedure including proteinase K treatment (see Methods). (C) Schematic of the long‐term degradation of origami structures and (D) percentage of intact origami structures, determined by gel electrophoresis, as a function of incubation time in the presence of cells at 37°C, with (triangle symbols) or without (disk symbols) the nuclease inhibitor monomeric actin (200 nm). Experiments are triplicated with error bars (mean ± SD) indicated when larger than the symbol size.

We first studied the origami assembly in the presence of human embryonic kidney (HEK) 293T cells, a widely used immortalized cell line, in a conventional 2D culture with DMEM supplemented by 10 vol% FBS and the two antibiotics penicillin and streptomycin. The cells were cultured at 37°C with 5 vol% CO_2,_ and a DNA cocktail coding for triangle origami (scaffold + staples) was directly added to cells after different culture times and confluences (Figure 3A,B). After two days of cell culture (15% confluence), the evolution of the morphology of the nanostructures was characterized as a function of incubation time with cells by AFM (Figure 3B top; Table S3). To enable accurate structural analysis of the folded DNA objects, we established a purification protocol that removed both excess staples and the high protein concentration present in the medium (Figure 1B right). The procedure consisted of polyethylene glycol (PEG) precipitation to eliminate staple excess, followed by proteinase K treatment and filtration prior to AFM imaging (see Methods). Notably, well‐folded structures were observed after only 5 min of incubation (*ρ =* 47%), with a yield remaining stable over 1 h, showing ultra‐fast formation of stable origami structures, as in pure DMEM (Figure 1C,D bottom) but in the presence of living cells. Similar results were obtained after 3 days of culture and with a larger amount of cells (45% confluence, Figure 3B bottom; Table S3), showing that the assembly was not much affected by the factors secreted by cells during their culture. To evaluate the generalizability of this in situ assembly method, the same experiment was repeated with HeLa cells. After 2 days of cell culture (44% confluence, Figure S6), well‐formed origami structures were also obtained after 5 min (Figure S7 and Table S3, *ρ =* 37 %), showing the versatility and robustness of the approach.

We next investigated the stability of in situ self‐assembled origami structures over prolonged incubation with HEK cells at 37°C (Figure 3C). Using agarose gel electrophoresis (Figures S8 and S9), we established the fraction of intact DNA origami structures over time and found that they remained stable for the first 24 h before starting to degrade (Figure 3D). This stability was prolonged to at least 3 days by adding the nuclease inhibitor monomeric actin [36] in the medium (Figure 3D). Moreover, during the incubation with the DNA cocktail, the HEK cells proliferated (Figures S10 and S11) and showed similar viability as in the absence of DNA (Figure S12), demonstrating that neither the presence of hundreds of staples, nor in situ self‐assembly into origami structures interfered with normal cell activity, confirming the well‐established DNA origami biocompatibility [18, 24, 25, 26, 27, 28, 29, 30].

**FIGURE 4:**
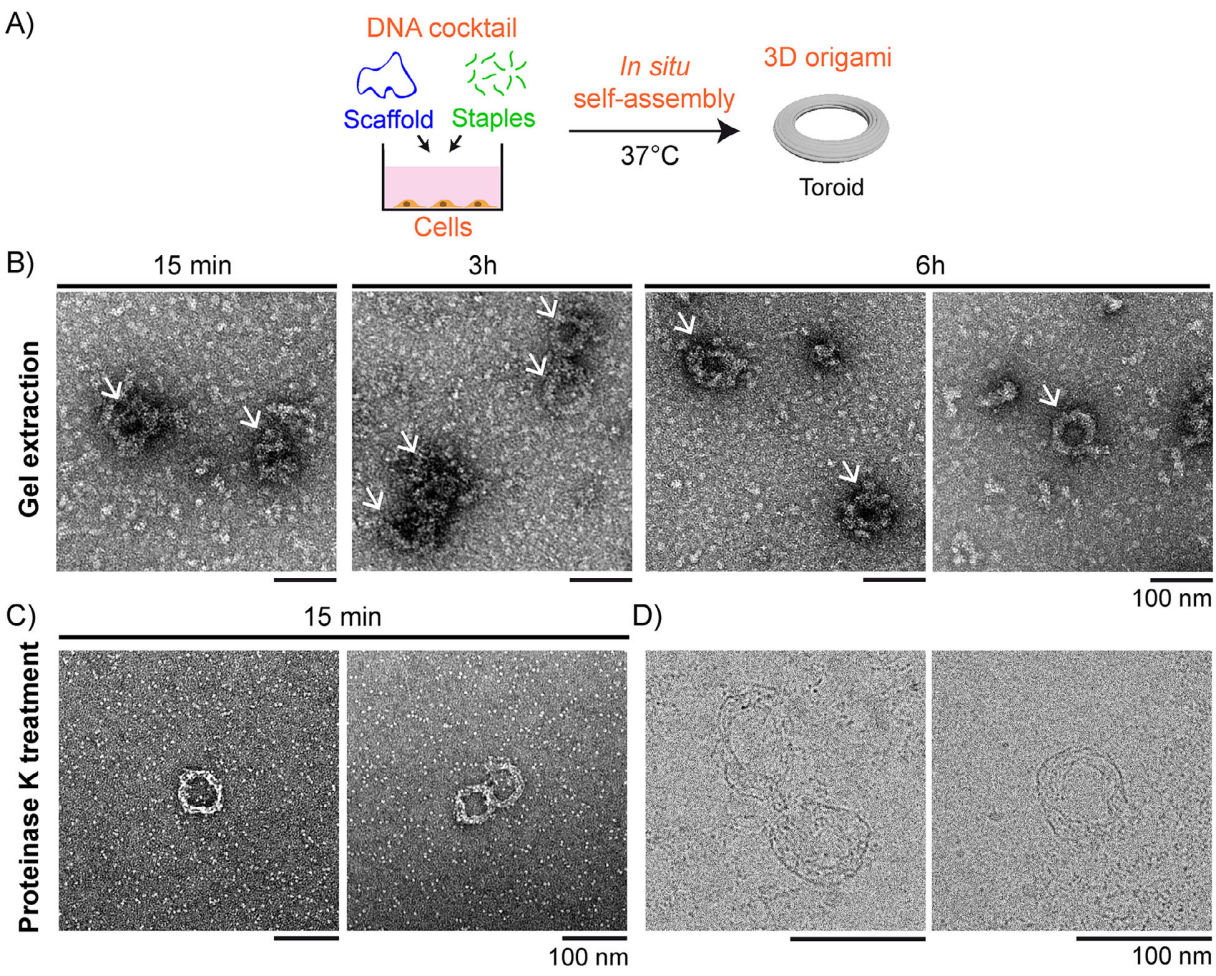
Fast self‐assembly of 3D origami structures in the presence of HEK cells in culture. (A) The experiment consists of adding a DNA cocktail coding for toroid origami ([scaffold] = 10 nm; [staple] = 100 nm) to cells in culture (DMEM, 10% FBS, Penicillin‐Streptomycin) and incubating the system at 37°C with 5 vol% CO_2_. (B) TEM images of toroid origami structures (white arrows) obtained after 15 min, 3 h, and 6 h of incubation at 37°C in the presence of cells. Samples were purified by band extraction from the agarose gel shown in Figure S13. (C,D) TEM (C) and cryo‐EM (D) images of toroid origami structures obtained after 15 min (C) and 1 h (D) of incubation with cells at 37°C. Origami samples were purified from proteins before imaging using a multi‐step procedure including proteinase K treatment. All scale bars are 100 nm.

We next challenged the possibility to assemble in situ more complex 3D morphologies, using a DNA cocktail coding for the 3D toroid design (Figure S5). The DNA cocktail was directly added to HEK cells after 1 day of culture (Figure 4A). Samples after different incubation times at 37°C with cells were purified by gel electrophoresis (Figure S13). TEM images of the extracted bands (Figure 4B) revealed toroidal structures with a rough surface attributed to the non‐specific adsorption of proteins [37] present in the medium, and with a diameter (77 ± 6 nm, *n* = 14) in agreement with the bare design (Figure S5). To get a better visualization of the formed origami structures, we applied the purification protocol involving proteinase K, as in Figure 3B. After 15 min only of incubation with cells, purified origami structures revealed a thinner toroidal morphology, showing successful removal of adsorbed proteins (Figure 4C). Notably, when cryo‐EM was performed on this system, clear toroidal structures, including some inner structural details, could be observed (Figure 4D) and were similar to those observed after assembly in the pure medium without cells (Figure 2C right). All these results show, for the first time to our knowledge, that complex DNA cocktails containing hundreds of synthetic strands can faithfully self‐assemble in the presence of live cells to produce user‐defined and stable 2D or 3D DNA origami structures. As observed in the absence of cells, the self‐assembly process remained remarkably fast as it was completed in minutes timescale.

### 2.3 In Situ Origami Assembly During Cerebral Organoid Formation

**FIGURE 5:**
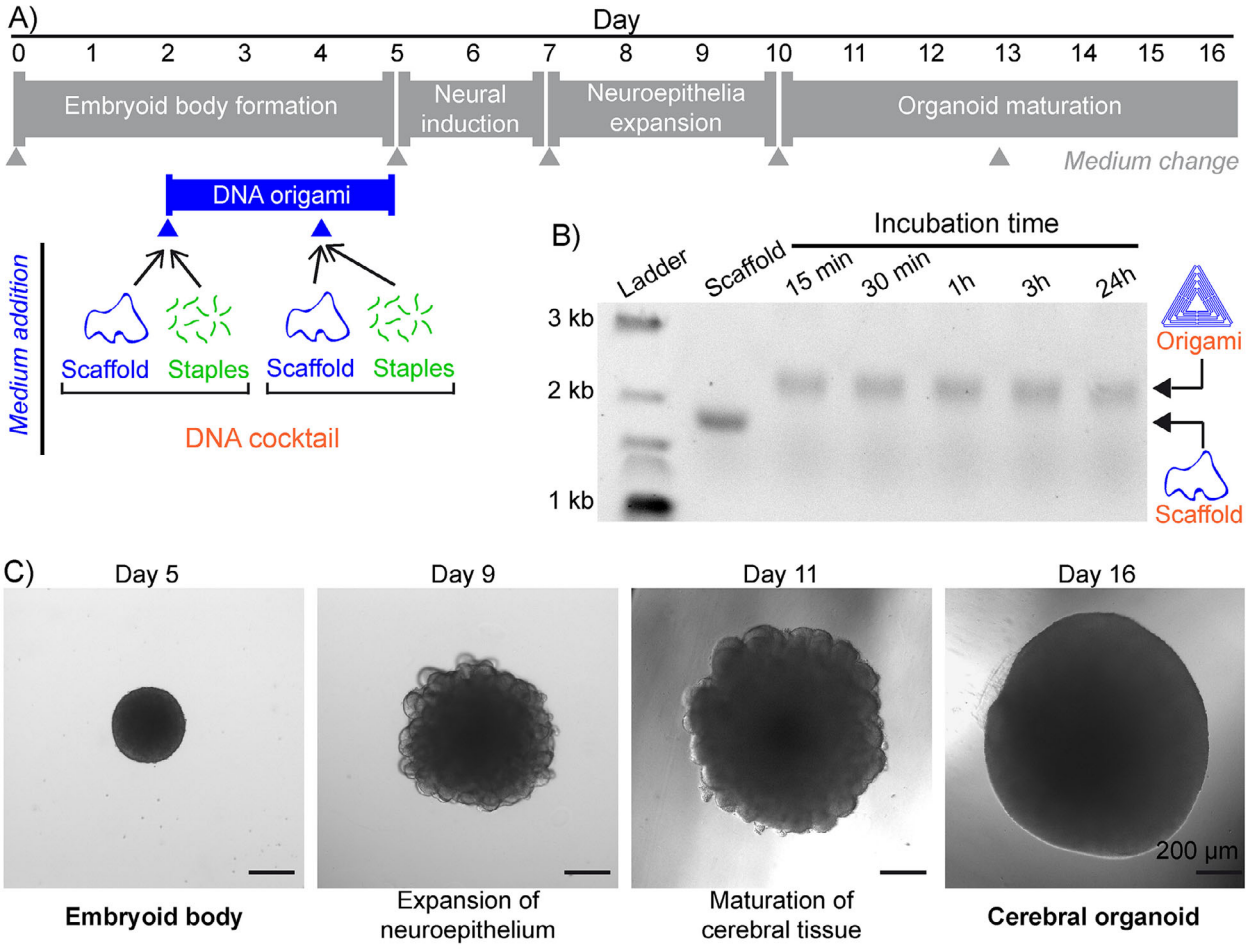
In situ self‐assembly of DNA origami structures during the formation of human cerebral organoids. (A) Top: protocol for cerebral organoid formation, with the different stages indicated on a grey background. Grey triangles indicate a change of the medium. Day 0 corresponds to human induced pluripotent stem cells (hiPSCs) seeding. Bottom: The medium supplemented by a DNA cocktail coding for triangle origami is added on days 2 and 4 (blue triangles). Final concentrations in the culture medium are [scaffold] = 1 nm and [staple] = 40 nm. (B) Electrophoresis agarose gel of the medium at day 2 as a function of time (15, 30 min, 1, 3, 24 h) after addition of the DNA cocktail. The first two lanes are the ladder and the scaffold alone, respectively. (C) Representative bright‐field microscopy images showing the evolution, after in situ self‐assembly of DNA origami structures, of an embryoid body (day 5) into a cerebral organoid (day 16). All scale bars are 200 µm.

Next, we explored the feasibility of in situ origami self‐assembly in the presence of a more complex 3D tissue‐like system. As a proof of principle, it was implemented during the formation of cerebral organoids from human induced pluripotent stem cells (hiPSCs), which is a multi‐step process involving different medium exchanges bringing the necessary factors for embryoid body (EB) formation (day 0–5), neural induction (day 5–7), neuroepithelium expansion (day 7–10) and organoid maturation (from day 10). A DNA cocktail coding for triangle origami was implemented during EB formation by a first addition of fresh medium containing all the DNA strands at day 2 (Figure 5A; Figure S14). Gel electrophoresis after different incubation times with EBs (Figure 5B) revealed DNA bands at a position corresponding to well‐folded triangle structures obtained in both cell‐free (Figure S15) and cell‐laden (Figure S16) media, showing successful origami self‐assembly in this complex system. Interestingly, origami structures were obtained in 15 min only and remained stable for at least a day. This process was repeated at day 4 of EB formation (Figure S14). Notably, at day 5, well‐formed EBs with smooth spherical shapes were obtained (Figure 5C left; Figures S14 and S17), showing that neither the addition of DNA strands nor the subsequent in situ self‐assembly of DNA origami structures perturbed the EB formation. Following their formation subjected to in situ origami assembly, EBs were differentiated by neural induction and evolved to bigger and raspberry‐shaped structures, indicating successful neuroepithelium expansion (Figure 5C; Figure S17, day 9), prior to maturation into structures becoming progressively larger and smoother (Figure 5C; Figure S17, day 11). At day 16, particularly smooth and well‐defined cerebral organoids were obtained (Figure 5C right; S17 bottom), showing that EBs subjected to in situ origami self‐assembly maintained their full capability of differentiation.

## 3 Conclusion

We have shown that direct mixing of a complex DNA cocktail (scaffold + staple mix) in a monovalent cation‐rich cell culture medium at 37°C enables the ultra‐fast, in situ self‐assembly into user‐defined elaborate 2D and 3D DNA nanostructures. The method was demonstrated to be particularly versatile and robust, functioning across a range of widely used biological media, including DMEM and RPMI (highly common for mammalian cell culture), Essential 8 (for stem cell culture), or PBS (involved in a variety of biological protocols). Unlike conventional DNA origami assembly methods, which require hours to days, this isothermal self‐assembly process was found to be extremely rapid, with well‐formed DNA origami structures being typically obtained in 5–15 min. As such, it can be seen as a convenient way to prepare DNA origami structures requiring nothing more than brief incubation at physiological temperature. But its most striking feature is that such a complex yet faithful self‐assembly is achieved in conditions compatible with live‐cell environments. This has allowed us to achieve the in situ self‐assembly of user‐defined 2D and 3D DNA origami structures directly in the presence of living mammalian cells, with nanostructures forming within minutes and remaining stable for a few days. The formation and presence of DNA origami structures did not perturb the cell behavior: HEK cells maintained normal growth and viability, while hiPSCs successfully formed embryoid bodies and differentiated into well‐defined human cerebral organoids. Mainly demonstrated here with DNA origami, this approach could be extended to other DNA assemblies, such as single‐stranded tiles (SST) [38] or DNA nanogrids [39], but may require sequence adaptation and/or longer assembly time, as achieved here with DNA nanotubes. As the method was functional with common cells (HEK and HeLa cell lines, commercially available hiPSCs) and in different formats (conventional 2D culture, 3D organoid formation), we envisage that the in situ DNA origami assembly described here is readily applicable to other cell types and tissues.

Considering the hundreds of DNA strands and thousands of base pairs to be properly combined, one may wonder how such an intricate isothermal assembly process can occur so efficiently in such a short time. A part of the answer may lie in the intrinsic properties of the DNA origami method and especially the use of a circular scaffold and a staple excess [12], which facilitates high‐yield folding even for complex 3D architectures [15, 16]. Our study further highlights the crucial role of staple excess and scaffold concentration as key factors driving rapid in situ self‐assembly. Devised by Paul Rothemund, the concept of DNA origami was published nearly 20 years ago in a breakthrough paper [12], which, by concretizing the seminal ideas of Nadrian Seeman [40], revolutionized nanoscience and many fields of research. Over the past two decades, the DNA origami method has proven to be not only exquisitely programmable but also exceptionally robust. Our findings further underscore this robustness, revealing that DNA origami self‐assembly naturally aligns with the ionic conditions optimized for biological function. The second part of the answer may lie in the fact that, although machine‐made, synthetic DNA has the exact same chemistry as biological DNA. Knowing that biological systems have evolved to conditions optimizing their functioning, which includes proper ionic conditions compatible with dynamic DNA assembly/disassembly, it is perhaps unsurprising that the media developed for cell culture and maintenance are also fortuitously optimized for ultra‐fast isothermal self‐assembly of DNA origami structures at 37°C. In this study, we have focused on origami made of structural DNA strands only, but the method is readily compatible with the incorporation of modified staples to enrich their functionality, for instance, toward the cellular interface. By enabling the formation of DNA origami structures in situ within live‐cell environments on a minute timescale, this method is the ground for the creation of a new class of environmental DNA nanomachines self‐assembling directly within biological systems to facilitate applications ranging from cell surface nanosensing to adaptive drug delivery and dynamic mechanobiological activation.

## Funding

This project has received funding from the European Research council ERC under the European Union's “HORIZON EUROPE Research and Innovation Programme (Grant Agreement No. 101096956)” (D.B.), the Institut Universitaire de France IUF(D.B.), and the Fondation pour la Recherche Médicale FRM No. ARF202209015925 (L.B.).

## Conflicts of Interest

The authors declare no conflicts of interest.

## Supporting information




**Supporting File**: smll72015‐sup‐0001‐SuppMat.pdf

**Supporting File**: smll72015‐sup‐0002‐MovieS1.avi

## Data Availability

The data that support the findings of this study are available from the corresponding author upon reasonable request.
