# Supplementary material for: Ultra‐Fast Isothermal Formation of DNA Nanostructures in Culture Media: Application to In Situ Assembly of DNA Origami With Living Cells
Source: Small. 2025 Dec 23;22(9):e09401. doi: 10.1002/smll.202509401 (PMC12895221; doi:10.1002/smll.202509401)
Supplement: Supplementary file 1 — Supporting File: smll72015‐sup‐0001‐SuppMat.pdf [file SMLL-22-e09401-s002.pdf]

Supporting Information

Ultra-fast Isothermal Formation of DNA Nanostructures in Culture Media: Application to *in situ* Assembly of DNA Origami with Living Cells

*Laura Bourdon, Gerrit David Wilkens, Samy Dehissi, Salammbô Hotte, Sergii Rudiuk, Mathieu Morel, Ayako Yamada, Gaëtan Bellot, Damien Baigl**

Contents

1. Materials
2. Methods
3. Supplementary figures S1–S17
4. Supplementary tables S1-S3
5. Legend of the supplementary Movie S1
6. Supplementary references

1) Materials

Materials

Single-stranded M13mp18 DNA scaffold was ordered from New England Biolabs at 100 nM (reference N4040S) and from Tilibit at 400 nM (named "p7249", reference M1-12), and p7560 scaffold was ordered from Tilibit at 400 nM (reference M1-32). The DNA scaffold were used as is without further purification. Oligonucleotide staples for 2D origami structures (triangle, rectangle and smiley shapes) were ordered from Sigma-Aldrich as 100 µM water solution and used without further purification. For the 3D toroid origami structures, the staples were ordered desalted from IDT. The staple mixes were used as is or concentrated by freeze-drying for experiments requiring high concentrations of staples (100 to 400 nM). DMEM 1× high glucose (HG) medium (Corning™, reference 10-013-CV) and FBS from Cytiva (HyClone™, reference SV30160.03) were ordered from Fisher Scientific. Powder DMEM HG (reference D5648) and MTT toxicology assay kit (reference TOX1-1KT) were purchased from Sigma-Aldrich. RPMI-1640 medium (Gibco™, reference 21875-034), PBS 1× (Gibco™, reference 70011-36), Essential 8™ medium (Gibco™, reference A1517001), Penicillin-Streptomycin antibiotics (Gibco™, reference 15140-122), Trypsin (Gibco™, reference 15400-054), Proteinase K (Thermo Scientific™, reference EO0491) and ethylenediaminetetraacetic acid (EDTA, Invitrogen™, reference 15575020) were purchased from Thermo Fisher Scientific. Actin protein from rabbit skeletal muscle was purchased from Tebu Bio (Cytoskeleton, reference AKL99). The DNA gel dye GrGreen 10 000× was supplied by Excellgen (Reference IV-1001). Rock inhibitor Y-27632 was ordered from Hello Bio (Reference HB2297). The cerebral organoids were formed using STEMdiff™ Cerebral organoid kit supplied by STEMCELL Technologies (Reference 08570). The HEK293T cell reference is 293T ATCC® CRL-3216™, HeLa cell reference is ATCC® CCL-2™ and Episomal hiPSC Line was supplied by Thermo Fisher Scientific (Gibco™, reference A18945). All other chemicals were supplied by Sigma-Aldrich.

Preparation of concentrated DMEM

A concentrated DMEM solution (2× or 5×) was prepared with DMEM high glucose (HG) powder and sodium bicarbonate, and were diluted with water and DNA solutions to reach a final composition of 13.4 $g.L^{-1}$ DMEM and 3.7 $g.L^{-1}$ sodium bicarbonate. The concentrated DMEM solution was filtered through a 0.22 µm pore-size membrane and stored at 4°C until use. This solution was used for all experiments, with the exception of the cell experiments and the one shown in **Fig. 1B**.

Preparation of 1× DMEM with or without supplements for Fig. 1B and cells experiments

1× DMEM HG solution was used as is, or supplemented with 10 vol% FBS, 100 U.mL^{-1} penicillin, and 100 µg.mL^{-1} streptomycin.

DNA nanostructure design and strand sequences

►For the 2D DNA origami structures, such as the sharp triangle and the smiley, we used the staple strand and M13mp18 scaffold sequences, as described in the 2006 article by P. Rothemund(1). For the 2D tall rectangle DNA origami structure, we used the staple sequences described in Rothemund article (1) but we removed staples from the short edges in order to prevent inter-origami stacking. The list of removed staples together with the reference used in the 2006 article by Rothemund (1) is given below:

t7r0f; TGAAAGTATTAAGAGGCTATTATT
t7r10f; AAAAGTAAAACGTCAAAAATGAAAAAACGATT
t7r12f; TTTTGTTTGCTTATCCGGTATTCTAAATCAGA
t7r14f; TATAGAAGACGCGCCTGTTTATCAGTTCAGCT
t7r16f; AATGCAGAGAAAAAGCCTGTTTAGGGAATCAT
t7r18f; AATTACTACATAGGTCTGAGAGACGTGAATTT
t7r20f; ATCAAAATGAAGATGATGAAACAAAATTACCT
t7r22f; GAGCAAAAACTTCTGAATAATGGATGATTGTT
t7r24f; TGGATTATGCCGTCAATAGATAATCAACTAAT
t7r26f; AGATTAGACCAGCAGAAGATAAAAAATACCGA
t7r28f; ACGAACCACTACATTTTGACGCTCACGCTCAT
t7r2f; CTGAAACAGTCAGACGATTGGCCTCAGGAGGT
t7r30j; GGAAATACCAGGAACGGTACGCCATTAAAGGGATTTTAGA
t7r4f; TGAGGCAGGCGTCAGACTGTAGCGATCAAGTT
t7r6f; TGCCTTTAAGACAAAAGGGCGACAGGTTTACC
t7r8f; AGCGCCAAGCAGATAGCCGAACAATTTTTAAG
t-7r10e; CATTCAACCTTGCCCTGACGAGAACATTCAGT
t-7r12e; AAACAGTTTAATGCAGATACATAAGAATACCA
t-7r14e; TTTTTGCGCAGAAAACGAGAATGAAATGCTTT
t-7r16e; TCAATTCTGATGGCTTAGAGCTTAAGAGGTCA
t-7r18e; AGGTAAAGACTAATAGTAGTAGCAAGGTGGCA
t-7r20e; AGAAAAGCATTCAAAAGGGTGAGATAATGTGT
t-7r22e; GATTGACCCCCAAAAACAGGAAGATGATAATC
t-7r24e; CAGCTGGCGTAATGGGATAGGTCAAAACGGCG
t-7r26e; GCATAAAGGAAAGGGGGATGTGCTTATTACGC
t-7r28e; GAGTTGCATGTAAAGCCTGGGGTGAGCCGGAA
t-7r2i; AATAATAAATAGGAACCCATGTACAGGGATAGCAAGCCCA
t-7r30e; ACCCAAATGCAAGCGGTCCACGCTCCCTGAGA
t-7r32e; CAAGTTTTTTGGGGTCGAACCATC
t-7r4e; CAGCGAAATTTTTTCACGTTGAAAGAATTGCG
t-7r6e; CGCCTGATGACAGCATCGGAACGAACCCTCAG
t-7r8e; GAATAAGGAAATTGTGTCGAAATCTGTATCAT

►For the 3D DNA origami structure with a toroidal shape, we used staple strand sequences designed with ENSnano, and a p7560 scaffold. The list of sequences is given below:

CTT CTG TAA ATC GTC GCT TAC ATA
CTG TCC AGT TTA ATG GTT T
CAA ACC GTT TAA AGA ACG TGG ACT GGG AGA G
AGC CAG TAA TAA AAA TAA G
GAG GCA TCC ATC CTA AGC AAA T
CCA ACA TGT AAT TTA GGC A
GAG ATA CAA ATT CTT ACC AGT
TCA TTG CGC TCA ACA GAA CGG GTA TTA ATT AGC GT
AGA TCT TTT GTC GTC TTT CCA CTA CAA CG
GCT GAT ATC AAT CAT ATG TAC CAT TTT TG
GAT ATT CTT CAA CAA AGC CCA ATA GG
GTC AAA TCA CCA AAG CAT TTT GTT
GCC TGA GTA ATG TGT ATT CAC GTT GAA AAT CT
AAT AAA AAG GCT CCA AAA GGC TAC AG
ACC CTC ATA TAT TTT ACA TTA ACA TCC AAT AAA
TTT TTA GAT TTA ATT GTA TC
TTC AAC GCA AGA AAG AAT TA
TTT GCG GGA GAA GTC TTA AAC AG
ATC GCG CAG AGG CCA AAA ACA ACA AT
GCT TTG GCA AAA GAA GAT GAT GTG AA
AAC GTC TGG CAA TTC ATC AAT
GAT TTT TTT ATT CCC TTA GAA TCC TTG AAA A
AAA TAA GGA TTA TAA GGT TAT CTA AAA
GGT TTT GAC ATA TCA AAA TTA TTA AGG GTT A
CAG ATA TCT TTC CAG AGC CTA ATT TGA ATT AAC
CAT TAC CGC GCC CAA TAG CAA TTT ACG A
GCA CTC ATC GAG AAC AAG CAA GAA ACG ATT
CCT GTA GCA TTC CAC AAA CCT ATT
AAC CTA GCG GAG TCT CTG AAT TTA C
GGA TAG CGT TTC AGC GGA GTG AGA ATA CAC CCT
ACC GCC ACC CTC AGA GCC ACG AAA GAG ACA
CTC AGA TTG CGA ATA ATA ATT TTG GTA
TCA GAA CCG CCA CCG TGT ATC ACC GTA CTC ACT TGA C
AAA GAG GAA GTT TCC ATT AAC ATA GG
AGG CTT TGA GGA CTC CAA AGC AAT
AGA CAG TAT CAG CTT GCT TTC GAG GTG
GCT TGC AGG GAG TTA AAA CCG ATA G
AAC CGA TAT ATT CGG TCG CTG AGA CCA AGC GCG
ATC CTT GTA ACA TTA TCA TTT CGC CTG ATT
ATT CGA CAA AAC AGA GGT GAG GCG TAT TAG TC
TAG ACT TCC ACC AGA AGG AGC GAT CGG G
GGA TTT CCT GCA ACA GTG CCA CGC T
AGC CGT CAA TAG ATC CTG A
AAC AAC TAA TAG ATT AGC AAA TGA AA
GGA GCA CTA TAA TCC TGA TTG TTT AGA AAT TGC GTA
AAT GAA ATA GCA ACA GTT GAA AGG ATG GTC AGT
AAT AAT AAC AAG ATT AGT TGC TAT TT
TCA GAG AGA TAA CCC ACC GAA CAA AGT TA
AGT CAG AGG GTA ATT GAA ACG CTA A
TGA ACA CCA AAA GAA CTG GCA TGT GTC CTT AGT GCT

TGC CCG TAG TCA AAA AAT AAA AGA
GCC TTC CCT GCC TAT TTC GGG ACA G
GGA GTG TAC ACA TGA AAG TAT TAA GTA CAA A
TTT GGC GCC AAA GAC AAA AGA CCA GTA
ATG GCT TGA GAC TCC TCA AGA GAA GG
TCC TCA TTA AAT TAT TCA TTA AAC CGC C
TGG CCT TGA TAT TTT GAT ATA AGT ATA GCC CGG AAT AG
AAG AAT TGA GAG GAG GCC GGA TAT TCA GCC TCC
CTG GCT GAC CTA ACA AAG CTG CTC ATT CA
GTA CAG ACC AGG CGA CGG GTA AAA TAC GTA A
CAA CTT TGA AAG AGG AGA AAC ACC AG
TCA TAA GGG AAC CGA ACT GAC AAA CGA AAG
GGA ACG AGG CGC AGA CGA ATT TCA ACT TTA AT
ACC TGC TCT GCG ATT TTA AGA AC
CCT GAT AAT ACA ACG GAG ATT TGT AAA TAC CGA CGA ACG TTA TTA CGC AT
TAG CCC TCA CCA GCA GAA GAT AAC TCG TAT TAA
TTT AAT GAT AAC ATC ACT TGC CTA AAA GAG T
TGG CAC AGA AAC TAT CTA AT
CAG AGA TAG AAC CCT TCT ATA TTA CCG
AGG GAC ATT TGC TGA ACC TCA AAT TAT CTT TA
ACA GTG CGG CCC TGT TAT TAA TGG ACC ATC TGT AA
AAC CCC GCT TCT AAT CTA ATC GGC TGA CGC
TGA CAA TGT CCC GGA GGA AAC GC
CTT CTA AGT GGT TGT GAA TTC
TCA GGA GAA GCC AGG GTG AAT ACA TAC ATA AA
ACG GCC AGT GCC GAG CTC GAA TTA AGG GG
CCC AGT CAC TAA GTT TAT TTT GTC ACG GGG TCA GT
GCC GTC ATA TGG TTT ACC AAT GAT ACA
GCA CCA TTT AAT CAG TAG CGA CAG AAG CCA TT
TGG GAA TAG CGT CAG ACT GTA GCT GGT G
GGT GAA TTA TCA CCG TCA CCG ACT TAA ATA TTG A
TCA GAG TTT CGG TCA TAG CCC C
AGA GCC ACC ACC CAC CAG AAC C
CTC AGA GCC AAT CAA AAT CAC CGG AAA GC
CGA GGC ATA AGA AGT TTT GCC AGA GGG G
CCA CAT TCA ACT AAT GCA GAT ACA TAA GTA AAT T
AGG AAT AGA TAG CGT CCA ATA CCC TGA CT
AGT TGA GAT TTC ATT GTG AAT
AAA ACG AAC TAA CGG TAC CAG TCA GGA CGT TG
TTA ACC GAC TTC TTT GAT TAG TAC GCG AAC TGA
CTG TCC AGG GCG CTA GGG CGC TGC GAT TT
GGA TTT TGC AAC AGG AAA AAC GCT CAT GGA AGT AAT AAA
TTA AAG TAC AGG ATC AAG TTT TTT
CGT TAG AAT CAG AGC CGC TCA
AAC CTG TCG TGT TCC TCG TGC TCC AGC TG
TTT CCA GAA TCG GCC AAC GCG CGC CAA CG
TCA CAT CGT ATT GGG CGC CAG GGA ACA AG
GGT GCT CTC CGA ACT CTG ACC T
GCC TGG TTT CTT TTC ACC AGT GTT GAG T
GAG CCG GGG CAA CAG CTG ATT GCC AAG AA
TGT TTC CAG GAT CCC CGG GTA CCA AGC TTT C
TGG CGA CGT AAT CAT GGT CAT AGC
CGG GCC TCT TCG CTA AAG GCG ATT AAG TTG GGT TTT
AAC TGT TGC GAT AGC AGC ACC GAC CAT TAG CAA G
CGC CAT TCA CGT GGG AAC AAA CGG CTC ATT T

CAC CGC TTC GCG TTT TCA TCG GCA T
ACA GTA TCG GCC TTT GCC ATC
ATT CGA GCT TCA ACC AGA GCC AGA CGA TAA AA
CTT CGT ACC TTT AAT TGC TCC GCA AAT
AAG CCC GAA AGA AGA GGC TTT TGC AAA GTA AGA G
TCA GAA GCA AAG CGG AGT AAA ATG TTT AGA CTG
ATT ATA GAG CTT AAT TCT GCG AAC GAG TA
CAT AAA TCA AAA ATC AGG TCT TTA CTG CGG AAT CGT C
AAA CGA GCC TCA AAT GCT TTA AAC AGA AAG GA
AAA GCA CAT GCA ACG ATA
GCA AAG AAC GCG AGA AAA CTA ATT ATG GA
ACT ACG TGA ACC ATC ACC CAA GCG CGT ACT
CGA TGG CCC ACG AGC ACG TAT AA
CGT CTC ATC TTC TGA AAA AAC
TCA AAG GGC GCC TAA AAC GAC GAC AAA ACC TTG
AGT CCA CTA GTG ATA AAT AA
GTT GTT CCA GTT TGG TGG TT
TAG CCC GAG AAT TAC TAG AAA AAG CCT GTT AAA
TCA ACT TCA CCG CCT GGC CCG CTC ACA ATT CCA CA
AAT CGG AGT TGC AGC AAG CGG TC
CTG TTT GAT GGT GGT TCC GA
TCA AAA CCC GTC GGA TTC TCG GCT GCG C
TTT AAC CAA TCG TAA AAC T
TTA AAT CAG CGG ATT GAC C
ATT AAC CGG TTG ATA ATC AGA ATC AAT AT
GGC GCG TGG CAA AGA TTC AAA AGG GTG AGA AAG
GGT CAA TAA CCT GTT TAG GTA GTA G
GAT TTA GTT TGA CCA TTA GAG GCA AGG CGA TAA AAA
AGT TGA AGC ATA AAG CTA AAT
GTG TCT GGA AGT TTC ATT CCT CAA
AAA TCA TAG ACC TGA AAT ACC
AAG AGT CAA TAG AAA CAA ACA T
AGA TTA AGA CGC TAT ATG TA
CAT AGA TCC AAT CGC AAG AGG GTC GAG GTG CCG T
AAC AGA TTA ATT TTT TCA AAT AT
AAT CAA TAT ATG TAA AAC AG
CAT GTT CAG CTA ATG CAG AAC GCT CCC GAC TTG CGG GA
ATA GAT AAG TTC TAA GAA CGC GAG GCG TTT TA
TCC TGA ACA AGA AAA ATA ATA TCT TTC G
GCA TGT AGA AAC CAA TCA ACG
GGC TGT CTG TTT TTA TTT TCA TCG TTA TTT AT
AAC GAT CCC TCA TAG ACC AAG TAC C
CTA AAG TAC AAA GGC TAT CAG G
GAC GTG GTA GGG TAA TAG GAA CGC CA
TGA ATT TTC GAG TTT CGT CAC CAG AGG C
ATT TTG CTA AAC AAC TAA CCG TTC TA
GGT TCA TCG GGA AGG CAC CAA CCT A
AAT TCC TTT ATG CAA AAT TAA GCA ATA
CTT GAT GGC CGC TTT TGC GGG A
TTG CGC CGT TAT GAC CCT GTA ATA CT
GAC AAC AAC CAT CGC CCA ATT TTA AAA GAA GTT ACA AA
TTT GAT GCC ACG AAC AAA ACA TCG CCA TTA AAT CAT CG
TGC GGA ACA AAG AAA TAC AAA CA
GAA TTA TCA TCA TAT TAA TAC ATT TGA
TTA TCA GAT GAA GAT GAA AAT TTC A

ATG GTG CAC GTG AGT GAA TTA AAC AA
GAA CCT ACA GCC TTA AAT GAG CAG AAG C
TGC ACC CGC GAA CCG CCT GTT TAT CAA CA
AGC TAC AAT TTT ATC CTG AAT CTT ACC GCG CTA ATA
CGA GCG TAG AAG GCT TAT CCG GTA
GCC AGT TAC AAA ATA AAC AGC CAT ATA GGA AT
CCC AAT CCC AGA GAG AAT AAC ATA AAA ACA G
TTT TGT TTA ACT AAA CAG TTA ATG CCG AGT AAC AG
ATT CTG AAT GGT AAT AAG TTT TAA CAA TCA
ATT AGG ATC ATG TAC CGT AAC ACT TGT ATG GG
CAG TAC CAG GCG GAT AAC ATT TTC AG
GTG CCG TCG AGA GGG CAC AAA CAA ATA AA
GGA GGT TTA GTC ATG ACT TTT TAC CGC CAC CC
TGC CAC TAC AAC GAG GGT AGC AAC GGA GCC
AGG CAA AAT CGT CAC CCT CAG CAG CGA A
GAA TAC ACT AAA ACA CTC ATC TTT GAC ACT TAG CC
AAA CAA AGA TTG TGT CGA AAT CCG CG
GTC AGT ATT AAC ACC GAG AAG TAT
GAG AGC CAA AGC GTA AGA ATA CG
AAT CTA AAG CAT CAC CTC TGG CCA A
ATC AAA CCC TCA ATC AAC CAG TCA CAC GAC CA
TAT CAT TGA GGA CTT CTG AAT A
TGG CAA ATC AAT AGC TAT CTT ACC AGA AAC
CCT TTT TAA GGT GAC TCT ATG ATA CCG
CAG ATA GCA AGA ATT GAG TTA AGC CC
CCA GAA GGA AAC CCC AAA AT
AAT AAT AAC GGA ATA CCC CTG AAC AA
GCA GTA TGG AAG CGC ATT AGA CGG GA
GTT AGC AAA CGT AGA AGA TGT T
GGT GGC AAC ATT GAA AAT AGC AGC CTT TAA AAT A
AAC GCA AAG ACA CCA CGG AAG ACG TTG TAA AAC G
ATA GAA AAT GAA ACG TCA CCC GCC AGG GTA AAA TG
TCA ACC GAC GTT CCA GTA AGC GTC ATA C
TTG AGG GAG GGA AGG TGA GCC ATT
CGG AAA GCC AGA ATG GAA AGC GCG GTT TTG CT
ACC ACC AGA GCC CCA CCC TC
CAT TGA CGC AGG TCA GAC GAT
TTA CCC AAA TCA ACG TTC ATC AAG AGT AAT
GTG AAT AAC AAC ACT ATC ATA AAT AGC G
GGC TTG CCC TGA CGA CAG ATG AAC GGT
AAC GAG TAC GCC AAA AGG AAT TA
GGG CTT GAG ATG GTT TGT CAA
TAC CTT ACA TGT TCC CCA GCG ATT AT
TGG CTC ATT AAA CAA CAT TAT TAC AG
GGA AGA AAA ATC AAT TTG TAG CTA CGT TAA T
GAA GAA CTC ACA ATA TTT TTG AAT GGC
GGC CTT GCT GAG TGT TTT TA
GTA ATA TCC AGA ACA GAC CTG AGC AG
CCA GCC ATT AGA CAG GAA CGG TA
ATA CCT ACA TTT TGA GGG AGC GCT TTG
ATC GTC TGA TAC ATT GGC AGA TTC A
TCG TCG GTG GGC ACG AAT GCC CGC
GCC TTG ATT TAC GCT CGC CCT GGA AAA AGT AAG
ATT TCA CAT AAA TCA TTC TAA TGA GTG AGC TAA C
CCT GGT TGA ATT GTC AAC CTT A

GGT GTA ATG AGT AAA CAG CAA CAT AC
GGC TTA TGC GCA CGA CTT AAG ATT AAG ACT CCT TAT TAC
AAG CTA CGT GGT GCT TGT TAC CTC
GAT AAA GAC GGT GTG TGA AAT TGT TAT
GAT GTG CTG CTT ACG CCA GC
AAA CCA TGG AAG GGC GAT CGG TGA TGT GAG
TGC CTT TTA GAG CCA GCA AAA TCG GCG ACA T
CTT ATT AGC GTC AGG AAG ATC GCA TCG TAA CC
TTT TCA TGC CAC CCT CAG AAC CGG CCG CCA G
ACC AAA CCC TCG TTT ACC AGA CCC ACC GGA ACC
GTA ATA TTG CAT CAT TTT TGC GGA TGG CTT AG
ATA AAG TAG AAA GAT TCA TC
TAT TCA TTG AAT CCC AAT GAC CAT GTT T
AGG GAA GAG CCG GCG AAC GTG GCG AGT TCA GA
AAG CGA AAG GAG CTC ACG CAA A
GCA AGT GTA GCA AGG GAG CCC C
GGT CCA GTG AGG CCA CCG AGT GAG TA
ACG CTG CGC GTA ACC ACC ACA CCC GCC AGA ATC CTG AGA
CGC CGC GCT TAA TGC GCC GC
ATG GTT TAA ACA GGA GGC CGA
CAT TAA TGT CGG GAG CAA C
GCG GTT TGT AAT TGC GTT GCG CTC ACT ATA GGG
AGA CGA AGC ATA AAG TGT AAA
CCT GAG AGC AAA ATC CCT TAT TAG TA
CAC GCT GGT TTG CCC CAG CAC AGC TT
TCA TCA ACC TGT AGC GGC GAA AAT C
CGA GTA ACA AAT AAA AGA G
GTA ATC CGG AAA CCA GGC AAA GCT CAA GTT
GGG ATA GGT CAC GTT GGT AAA ATT CGC
GTA GAT GGG CGC ACT CCA GCC AGC TTT CCG G
GTG CAT CTG AAT TGT AAA CGT TAA TCC CAA AAA CAG
CCA GTT TGA GGG ACC GGG AAC CAG GAC GAC G
AAG CAA ACT CCA ACT TTT CAT TTG G
AGG ATT AGA GAA AAT ATC GCG TTT TA
GAG GTC AAA AAG ATT AAG AGG
ATA ATG CTG TAG CAT ATA AC
TAA ATA TGC AAC TAA AGA GAG ACT
ACC TTT TTA ACC TCC GTT TAT CA
GCT TAA GAG CTT GAC GGG GAA A
GGT TGG GTT ATA TAA CTG AG
AAT GCT GTA AAT CGG AAC CCT A
ATT TTA GTT AAT TTA TCA GGG
GAA ATA CCG AAG GTA AAG TAA TT
GGC GTT GAG AAT ATA AAG TAC CGA
AAT AAA CAC CGG AAT CAT ATA GGG
TCA TAT GCG TTA ATC GCC ATA TTT AAC AAT AAT C
GAG CAA ACT TCG CGT CTG GCC TTC ATT AA
AAT CGA TGA ACC TAT TTT TGA G
AGC ATG AAT TAA TGC CGG AGA GTA GTA AA
GAA GAG CCG GGA ACA ACT AAA GGA A
TTG TAT AAG CAA AAG GAG CTG AAA TAT TTA
TCA ATT CTA CTA ATA CTA TAA GGT C
TCA TAC ATA CAT TTC TTT TGA TAA
AAG CCT CAG TTC CCA ATT GCT GAA T
CGG TTG TAC GAA TTA TTC ATT TCA ATT GTC TGT ACG

CAA GAA AGA AAC AAT AAC GGA TT
AAC AAA ATT AAT TAC GCT T
ATT TAA CTA TAC AGT AAC AGT ACC TTT TAC
TTT GAA TTA CCT TTC AGG TTT
AGC CTT CCT TAT CAT TCC AAG TAG GGC TTA ATT
ATA AAG CCA ACC TGA GAG TCT G

►For the DNA nanotubes, we used a tile motif assembling through sticky ends of 7 nucleotides, which was designed by Elisa Franco's team (2). Compared to the original motif, the toehold on the 5' end of the oligonucleotide 7bSE2 has been removed. The list of sequences is given below:

7bSE1; TCAGTGGACAGCCGTTCTGGAGCGTTGGACGAAAC
7bSE2; TGTCTGGTAGAGCACCACTGAGAGGTAC
7bSE3-Cy3; CCAGAACGGCTGTGGCTAAACAGTAACCGAAGCACCAACGCT
7bSE4; CCAGACAGTTTCGTGGTCATCGTACCTC
7bSE5; GATGACCTGCTTCGGTTACTGTTTAGCCTGCTCTA

2) Methods

***In situ* self-assembly of DNA origami structure without cells**

The self-assembly medium, supplemented by water when concentrated, was first heated at 37°C using a ThermoMixer® (Eppendorf®) prior to direct introduction of the desired volume of a concentrated staple mix (400 nM to 4000 nM) without thermal pretreatment. After briefly vortexing the solution, a desired volume of concentrated scaffold solution (100 nM to 400 nM) was added and gently mixed by pipetting and tapping. The solution was shortly centrifuged, and kept at 37°C to start the incubation time. Incubation time for DNA self-assembly was stopped when the sample was fixed on a surface for imaging (AFM and TEM) or quickly cooled by introducing the solution into ice and liquid nitrogen prior to storage at -20°C until use. For all experiments in DMEM (except **Fig. 1B**), concentrated DMEM (2× or 5×) was used as a concentrated medium and its final concentration was 1×. For experiments in other medium (**Fig. S2**), 89 µL of 1× medium was used and supplemented with 10 µL of concentrated staples and 1 µL of concentrated scaffold. The final DNA concentration in each sample is indicated in the figure legends.

***In situ* self-assembly of DNA nanotubes without cells**

The concentrated DMEM was supplemented by water and heated at 37°C using a ThermoMixer® (Eppendorf®) prior to direct introduction of the 5 concentrated oligonucleotide solutions without thermal pretreatment, to reach a final concentration of 500 nM of each oligonucleotide. After vortexing the solution and short centrifugation, the solution was kept at 37°C overnight.

Fluorescence microscopy of DNA nanotubes

We used a Zeiss Observer Z1 microscope equipped with a Pan-Apochromat 100x/1.4 NA oil objective to observe DNA nanotubes (labelled with a Cy3 dye) formed in DMEM at 37°C. For the observation, the DNA nanotube solution was diluted 100-fold in a salt buffer (Tris-acetate pH 8, 12.5 mM $MgCl_2$) before being deposited on a glass cover slip. The nanotubes adsorbed to the surface allowed for in-plane observation (**Fig. 2D, *left*),** while the free nanotubes for volume observation (**Movie S1**).

Agarose gel electrophoresis

1 wt% agarose gel was prepared with GrGreen (0.8×) DNA gel stain in TBE 0.5× buffer containing 10 mM of $MgCl_2$. 15 µL of each DNA solution, such as the ladder (1kbp plus DNA), the scaffold in the DMEM HG (1 or 10 nM), and the DNA cocktail in the cell media, was mixed with 3 µL of loading dye solution (Gel loading dye 6×) and kept on ice prior to their loading in the gel. The electrophoresis was run at 50 V (BioRad PowerPac™ HC) for 3 h in an ice bath. The gel was analyzed (Syngene G:Box) to assess the stability of DNA origami structures with cells, or to purify DNA origami rings for TEM imaging. In this last case, the bands of interest were cut for DNA extraction using spin columns (Montage DNA gel extraction kit from Merck).

AFM observation of DNA origami structures

High-resolution environmental AFM was used to characterize the DNA assemblies. DNA assemblies in DMEM at 37°C were fixed at room temperature on a freshly cleaved mica surface (Nano-Tec V-1 grade Muscovite, Micro to Nano innovative Microscopy Supplies) that was pre-treated with 20 µL of 0.1 M spermine tetrahydrochloride solution for 10 min before being washed with deionized water and a buffered saline solution (Tris-acetate pH 8, 100 mM NaCl). 15 µL of DNA solution was deposited on the treated mica and left for 10 min adsorption. For DNA samples prepared with cells and purified according to the protocol including proteinase K treatment, the entire solution (50 µL to 70 µL) was deposited on spermine-treated mica and left for 20 min for adsorption. The solution was blotted off and the mica surface was gently rinsed with the buffered saline solution, and kept moist in this solution. The samples were observed with a Cypher ES AFM Microscope (Oxford Instruments) in tapping mode with 17-45 kHz resonance frequency in liquid and 0.09 N/m force constant tip (BL-AC40TS, Olympus), using the blueDrive™ photothermal excitation mode. Raw images were subjected to polynomial background subtraction, plane level correction, rows alignment using various methods and horizontal scars correction in Gwyddion.

Transmission electron microscopy

The DNA self-assemblies were fixed on a plasma-treated 200 mesh copper grid which supports a carbon film (Ted Pella, Inc.): 10 µL of DNA solution was added on the grid and left for 3 min before being blotted with filter paper. The sample was negatively-stained using uranyl acetate (UA): 5 µL of 2 wt% UA water solution was added and quickly blotted, then 15 µL UA solution was again added and left for 1 minute before a last blotting. The samples

were observed using a JEOL microscope at 200 kV (toroid origami structures self-assembled in DMEM), or a Tecnai Spirit microscope at 120 kV (toroid origami structures self-assembled in the presence of HEK cells).

Cryo-electron microscopy

DNA nanotubes formed in DMEM at 37°C without purification, and DNA toroidal origami structures formed *in situ* with HEK cells and purified by the multi-step protocol involving proteinase K were observed by Cryo-electron microscopy (Cryo-EM). 4 µL of the DNA samples were deposited on glow-discharged carbon-Formvar lacey grids (Ted Pella, USA), blotted from the back side, and flash frozen in liquid ethane with an EM-GP2 Leica plunger at 80% humidity. Cryo-EM images were acquired with a Glacios cryo-electron microscope (Thermo Fisher, USA) operating at 200 kV with a falcon IV camera and in low dose mode.

Cell culture and *in situ* DNA origami self-assembly

HEK and HeLa cells were cultured in 1× DMEM HG supplemented with 10 vol% FBS, 100 $U.mL^{-1}$ penicillin, and 100 $\mu g.mL^{-1}$ streptomycin, at 37°C and 5% CO_2. The cells were passaged every 3 to 4 days at ~80% confluence using 2× Trypsin solution and were seeded at 1.10^5 $cell.mL^{-1}$. For the *in situ* DNA origami self-assembly experiments, we seeded 100 µL HEK and Hela cells in 96-well plates, then proceeded with the *in situ* DNA origami self-assembly 24 h and 48 h later, respectively. A volume of culture medium corresponding to the total concentrated DNA volume to be added was first withdrawn, before addition of 10 µL (triangle, final concentration 400 nM) or 2.5µL (toroid, final concentration 100 nM) concentrated staple solution and gentle mixing by pipetting. 2.5 µL of concentrated scaffold (M13mp18 for triangles or p7560 for toroids, final concentration 10 nM) was rapidly introduced and gently mixed by pipetting and tapping the cell plate, before being re-incubated at 37°C to start the incubation time. Incubation time was stopped when the DNA sample (cell media containing the DNA cocktail) was withdrawn from the cell culture well and quickly cooled down by introducing it into ice. The DNA sample was stored at -20°C until further analysis and/or treatments such as agarose gel electrophoresis, purifications, AFM, TEM or Cryo-EM. During the experiments, the cells were observed by transmission optical microscopy using an Olympus CKX53 microscope and EP50 camera.

hiPSCs were cultured at 37°C and 5% CO_2, on vitronectin-coated dishes in Essential 8™, or in mTeSR™ (from STEMdiff™ Cerebral organoid kit) for the differentiation into brain

organoid. They were detached every 3 to 4 days at 80% confluence using 0.5 mM EDTA solution, and seeded in their cell culture medium supplemented with 10 µM Y-27632 after 10 or 5-fold dilution. For the formation of embryoid bodies, 9 000 iPSCs in 100 µL medium were first seeded in an ultra-low attachment V-bottom 96 well plate (Greiner Bio-One) and kept in culture for 5 days, as indicated in the STEMdiff™ Cerebral Organoid kit protocol. At day 2 after the seeding, a mixture of 78 µL of fresh medium, 20 µL concentrated staple solution and 2 µL of concentrated scaffold solution was added. This was repeated at day 4 with a mixture of 89 µL of fresh medium, 10 µL of concentrated staple solution and 1 µL of concentrated scaffold solution. In both cases, the final concentration of scaffold and the staples in the resulting cell medium was 1 nM and 40 nM, respectively. The differentiation of the embryoid bodies was initiated at day 5 and carried out until day 16 in a cell-repellent tissue culture dish (Greiner Bio-One), as indicated by the kit supplier. The formation of brain organoids was characterized by bright-field imaging using a Leica DMi1 microscope and Leica MC170 HD camera.

Determination of cell confluence

The HEK and HeLa cell confluence was determined by transmission optical microscopy image analysis using Image J software. A freehand line was drawn around the cell islets to determine their surface area. The confluence corresponds to the sum of the surface area islets compared to the total surface area of the image.

Purification of *in situ* assembled DNA origami structures formed with cells

To characterize the yield of DNA origami structures self-assembled with HEK or HeLa cells by AFM, and to characterize the morphology of origami rings by Cryo-electron microscopy (Cryo-EM), the cell media containing the DNA nanostructures were purified using a multi-step protocol. The process includes i) two cycles of polyethylene glycol (PEG) precipitation to remove free staples, ii) a proteinase K treatment to remove proteins, and iii) two cycles of filtration to concentrate the nanostructures and remove digested proteins. The cell media, thawed and kept cold on ice, were first mixed with a concentrated salted PEG solution (final concentration 4% PEG 8000, 500 mM NaCl), and was left for 15 min at room temperature after mixing the solutions. The solutions were centrifuged at 15 000 rcf for 15 min. The supernatants were carefully removed, leaving approximately 10 µL at the bottom of the tube, which was mixed with 40 µL of DMEM. This PEG precipitation protocol was repeated a second time. In order to degrade proteins from the solutions, proteinase K was introduced in

the solutions to reach a final concentration of 1 mg.mL^{-1}. The solutions were incubated at 37°C overnight, before being diluted in DMEM to reach a volume of 500 µL, and being purified two times on the same 100 kDa AMICON® ultra centrifugal filter using 2 000 rcf speed, each time.

Determination of the fraction of perfectly folded DNA origami structures

The fraction of perfectly folded origami nanostructures was determined by counting the number of objects presenting 3 sharp corners considered as perfectly folded triangle origami structures, and the number of objects with no sharp corner, or only 1 or 2. The count was performed using Image J software, and AFM images providing a field of view sufficient to observe a large number of objects and to distinguish their shapes (2 µm per 2 µm)

Stability of *in situ* assembled DNA origami structures with cells

To determine the stability of *in situ* assembled DNA origami structures in the presence of HEK cells, we introduced the DNA cocktail inside the culture well containing cells seeded at 1.10^5 cell.mL^{-1} the day before (10^5 cells per well), and left for 1h, 3h, 6h, 24h and 48h with the cultured cells. For each incubation time, the cell medium containing the DNA cocktail was collected and placed on ice before being stored at -20°C. To increase the stability of the DNA origami structures with cells, 200 nM of monomeric actin, acting as a nuclease inhibitor, was added to the cell culture medium 1 h after addition of the DNA cocktail. In this case, the DNA origami structures were left for 4 h 40 min, 8 h 20 min, 24 h, 48 h and 72 h with cells in culture.

Electrophoresis DNA agarose gels were used to quantify the degradation of *in situ* self-assembled DNA origami structures in the presence of HEK cells. We identified DNA bands corresponding to well-formed origami structures and free staples in cell media, and assumed that the DNA detected between these two bands corresponded to degraded origami structures (**Fig. S8-S9**). Using Image J, we defined and plotted the lanes integrating the band of well-formed origami and the one attributed to degraded origami structures, for each incubation time (**Fig. S8-S9**). The percentage of full DNA origami structures in the cell medium was calculated from the amounts of well-formed origami structures and degraded origami structures determined by peak area integration of fluorescence intensity.

HEK MTT assay

The proliferation assay was performed using a MTT toxicology assay kit. The cell culture media were replaced by serum-free cell culture media containing 10 vol% MTT reagent and introduced to each cell culture well and to another one without cells (control), before maintaining the plate at 37°C for 3 h incubation. 100 μL of MTT solubilization solution was added in each well, and the plate was gently mixed at room temperature for 15 min, protected from light. A microplate reader (Biotek Synergy HT) was used to measure the absorption of the solutions at 570 nm, and at 690 nm for subtraction.

3) Supplementary figures

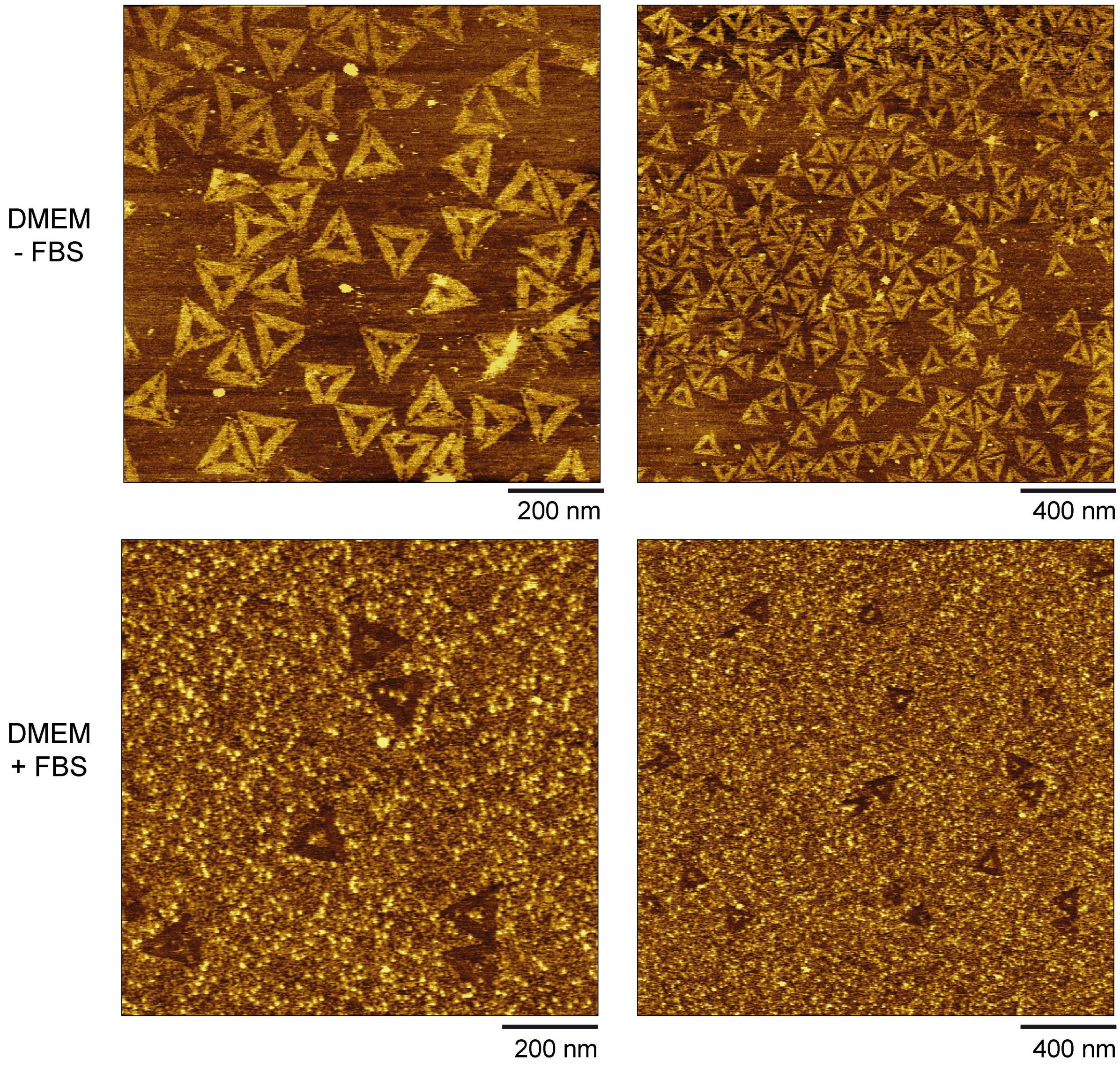

Figure S1. AFM images of triangle DNA origamis self-assembled at 37°C for 6 hours in DMEM without (top images) or with 10 vol% FBS (bottom images). [scaffold] = 10 nM; [staple] = 100 nM. DNA origamis were purified by agarose gel electrophoresis (see gel in **Fig. S15**) prior to AFM imaging.

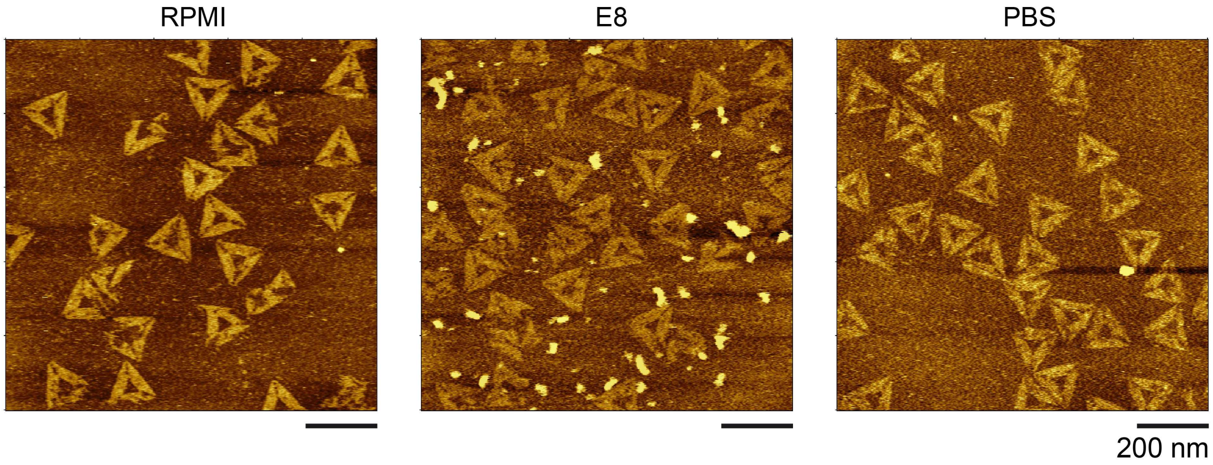

Figure S2. AFM images of triangle DNA origamis self-assembled at 37°C for 3 hours in RMPI (*Roswell Park Memorial Institute*), E8 (Essential 8^{TM}) or PBS (Phosphate Buffered Saline). Origamis were observed directly after assembly and without purification. [scaffold] = 1 nM; [staple] = 40 nM.

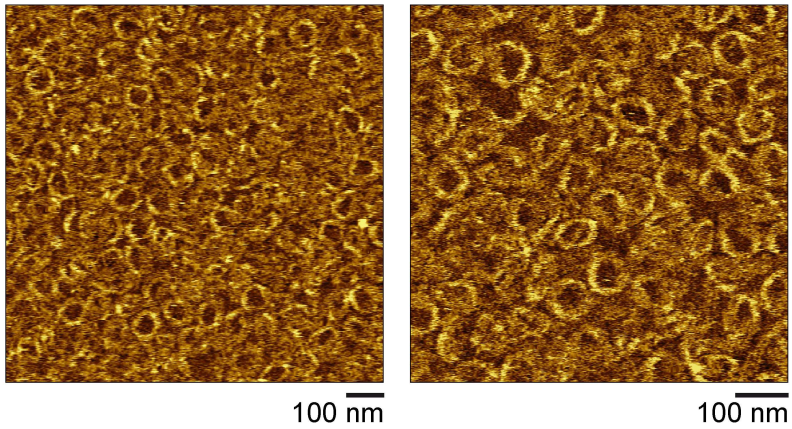

Figure S3. AFM images of toroid origamis self-assembled at 37°C for 15 min in DMEM. Origamis were observed directly after assembly and without purification. [scaffold] = 10 nM; [staple] = 100 nM.

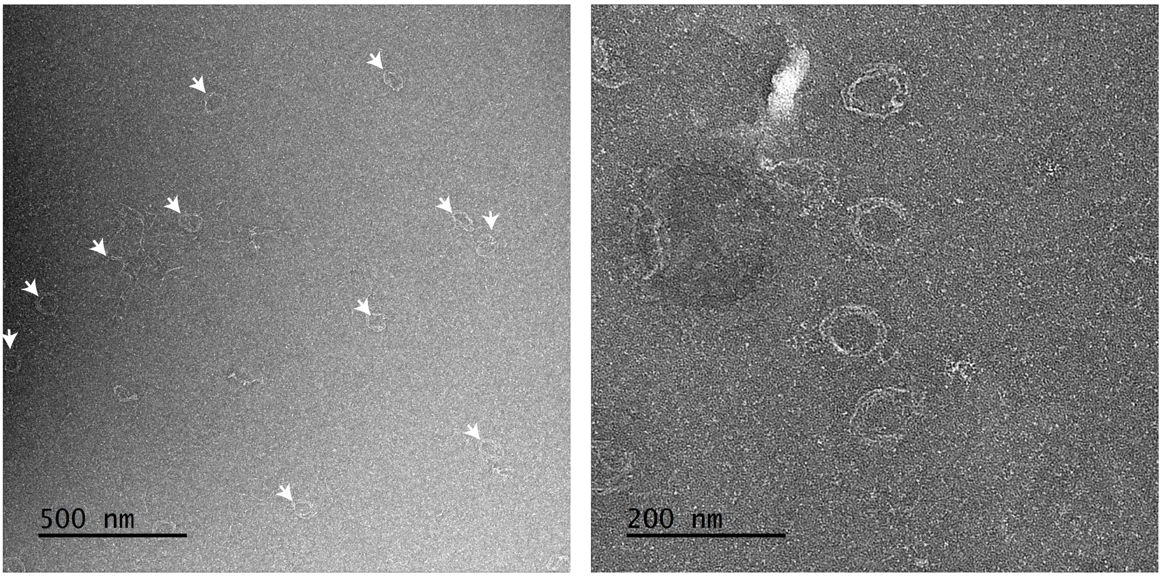

Figure S4. TEM images of toroid origamis self-assembled at 37°C for 15 min in DMEM. On the left images, well-formed toroids are pointed by arrows. DNA origamis were purified by agarose gel electrophoresis prior to TEM imaging. [scaffold] = 10 nM; [staple] = 100 nM.

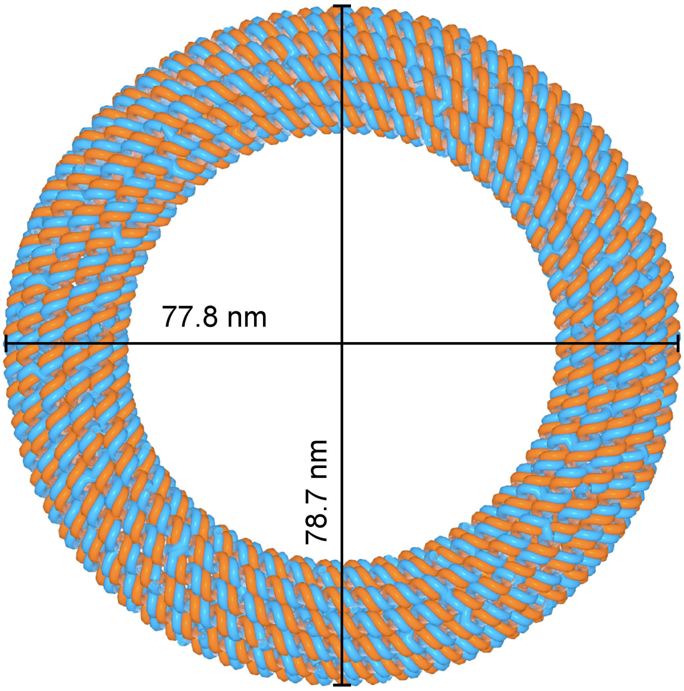

Figure S5. Toroid DNA origami design and dimensions.

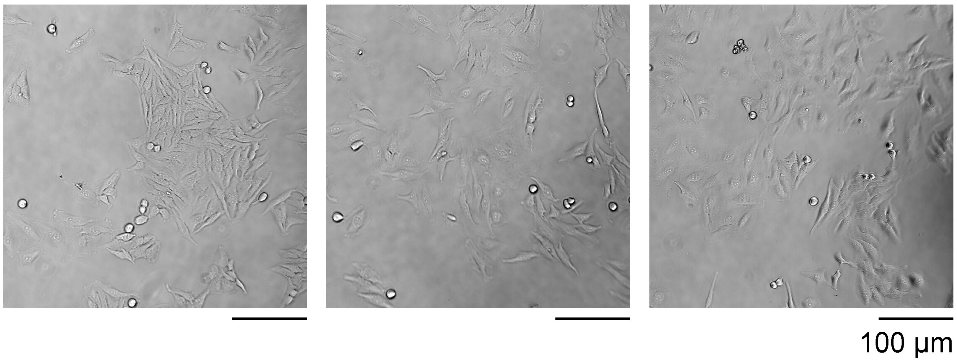

Figure S6. Transmission optical microscopy images of HeLa cells after two days of culture and *in situ* self-assembly of DNA origami structures.

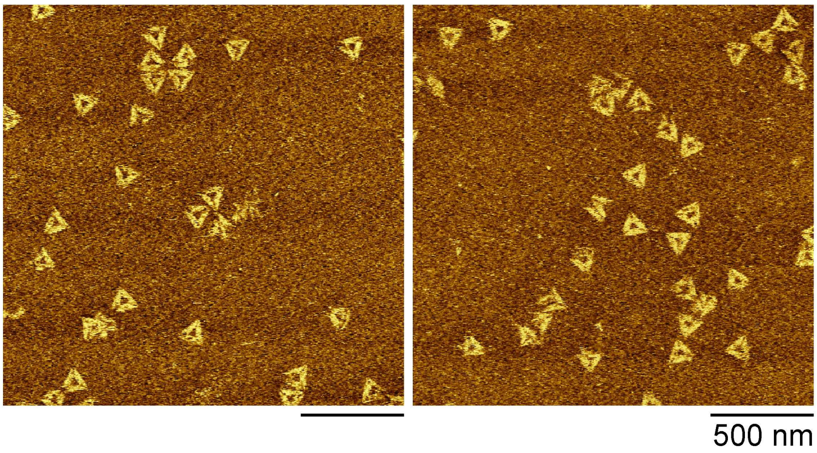

Figure S7. AFM images of triangle origami nanostructures self-assembled with HeLa cells for 5 min. Origami structures were purified using a multi-step protocol including proteinase K treatment before imaging. [scaffold] = 10 nM; [staple] = 400 nM.

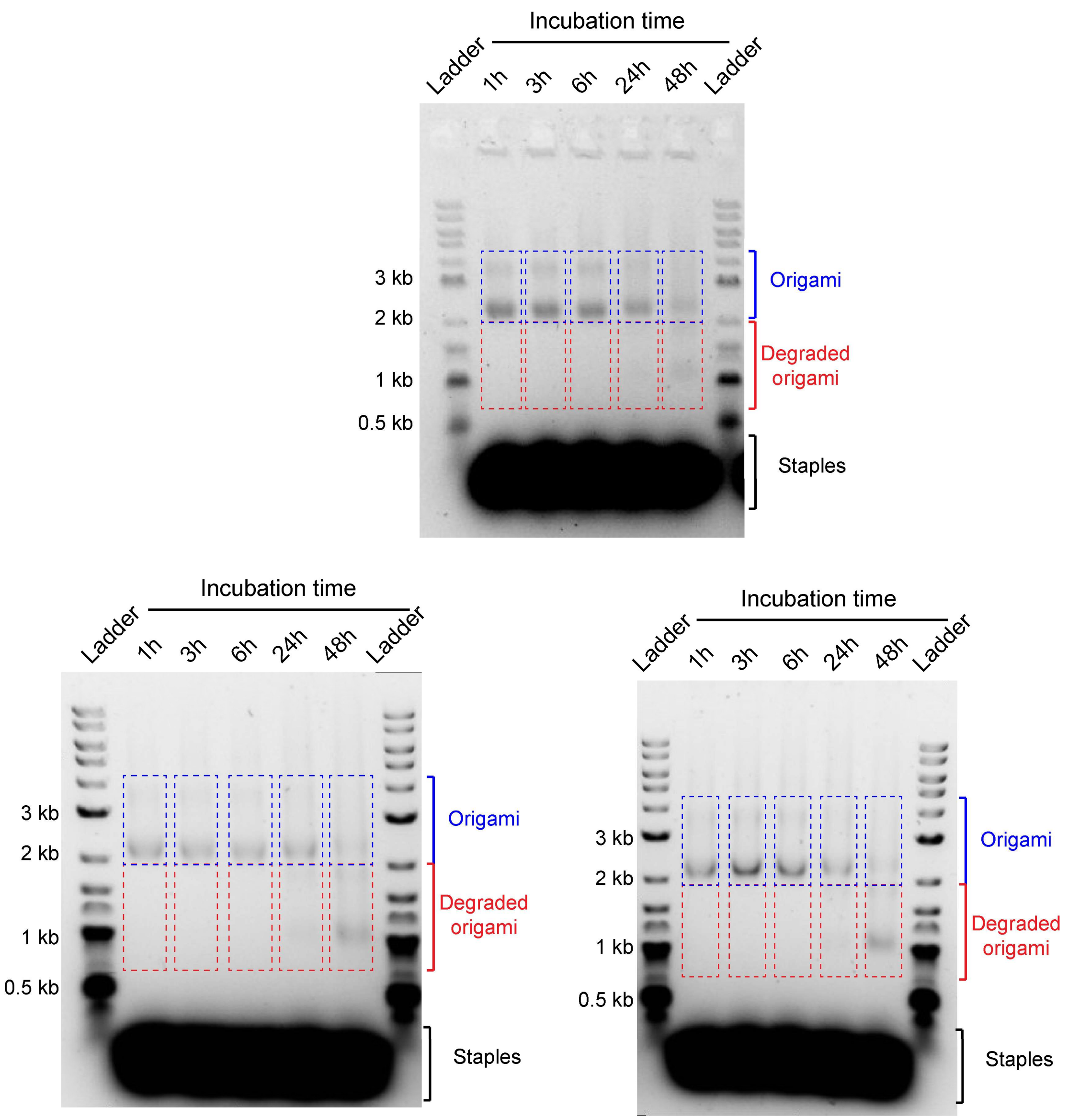

Figure S8. Electrophoresis agarose gels used to quantify the percentage of intact and degraded DNA origami structures as a function of incubation time, in the presence of HEK cells at 37°C (Fig. 3D). The dashed squares show the areas analysed for the quantification (see Methods). Each gel represents an independent experiment.

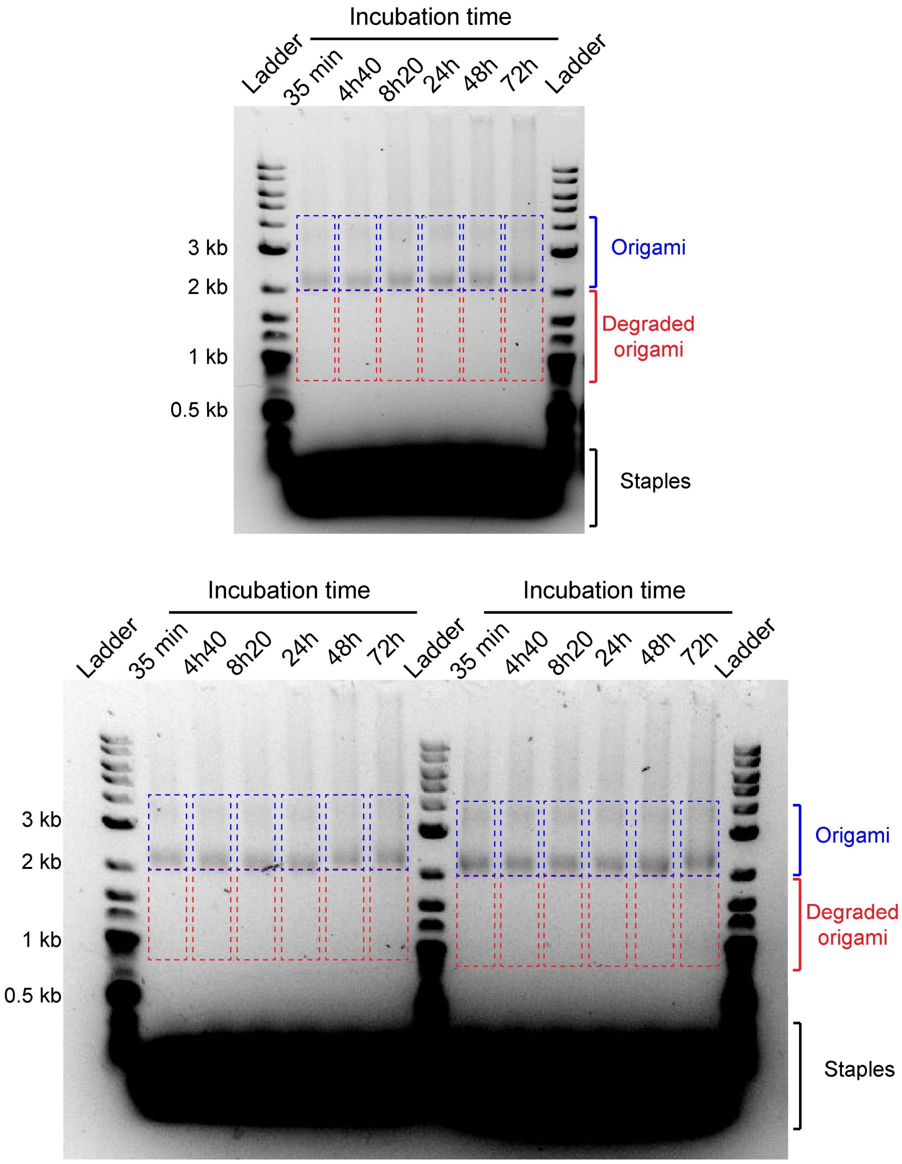

Figure S9. Electrophoresis agarose gels used to quantify the percentage of intact and degraded DNA origami structures as a function of incubation time with HEK cells at 37°C and nuclease inhibitor monomeric actin (200 nM, Fig. 3D). The dashed squares show the areas analysed for the quantification (see Methods). Each gel represents an independent experiment.

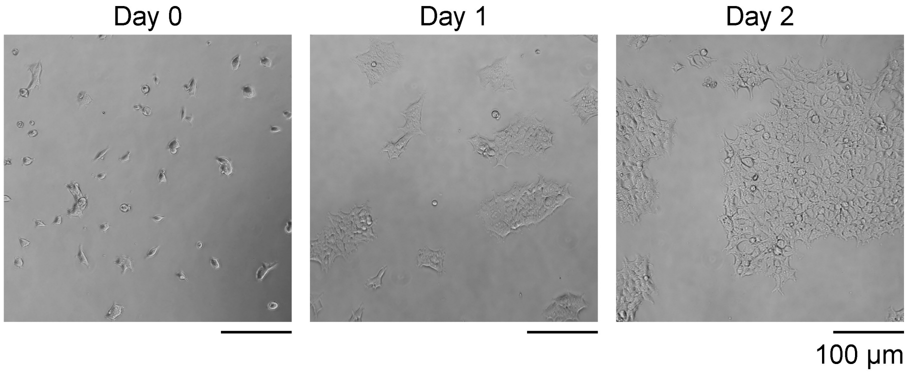

Figure S10. Transmission optical microscopy images of growing HEK cells in the presence of the DNA cocktail coding for triangles ([scaffold] = 10 nM; [staple] = 400 nM) introduced at day 0.

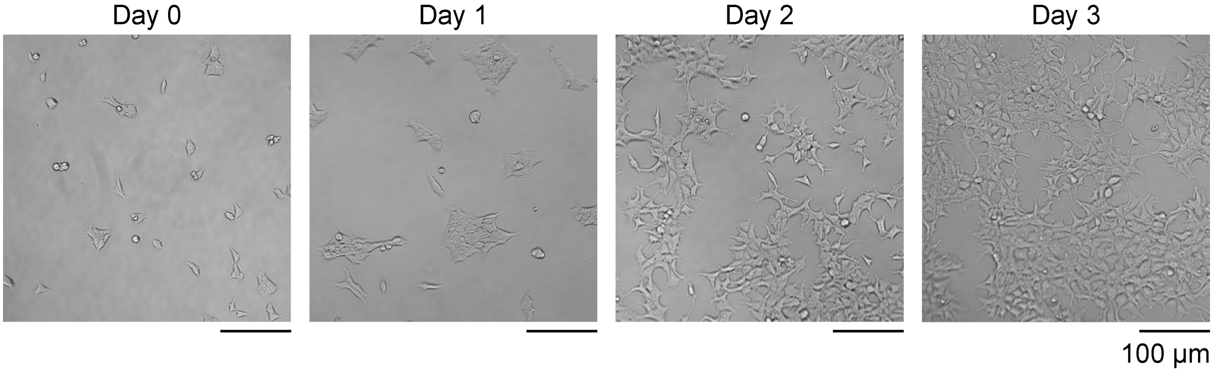

Figure S11. Transmission optical microscopy images of growing HEK cells in the presence of the DNA cocktail coding for triangles ([scaffold] = 10 nM; [staple] = 400 nM) and nuclease inhibitor monomeric actin (200 nM) introduced at day 0.

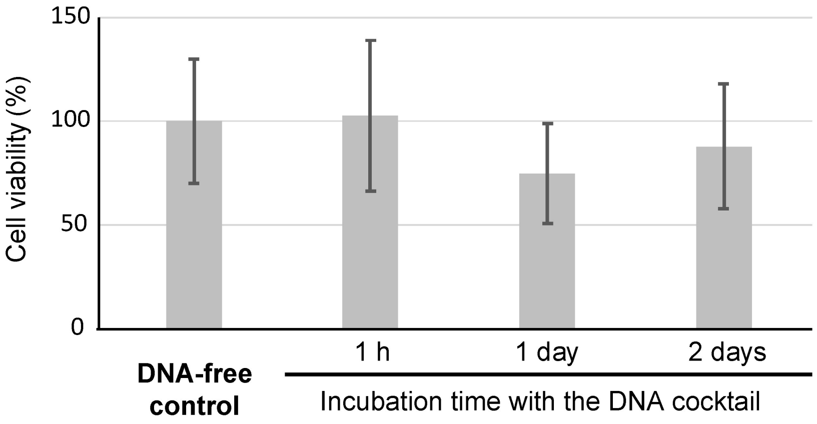

Figure S12. HEK cell viability (mean ± SD on triplicates) determined by MTT assay, of cells cultured in a DNA-free culture medium or containing the DNA cocktail coding for triangles ([scaffold] = 1 nM; [staple] = 40 nM). The DNA cocktail was left for 1 h, 1 d or 2 d with cells. The cell viability was normalized to DNA-free controls.

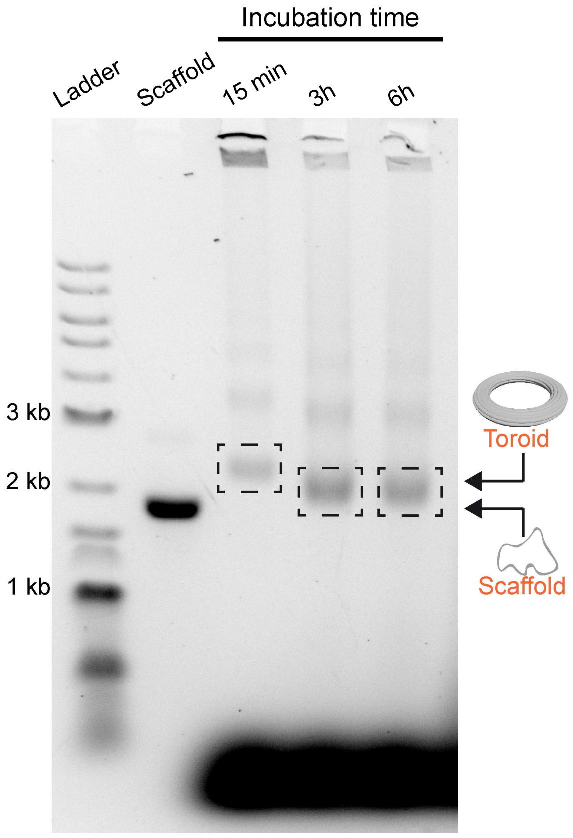

Figure S13. Electrophoresis agarose gel of DNA origami ring structures formed in the presence of HEK cells for 15 min, 3 h and 6 h. The dashed squared indicate the extracted bands observed by TEM in Fig. 4B.

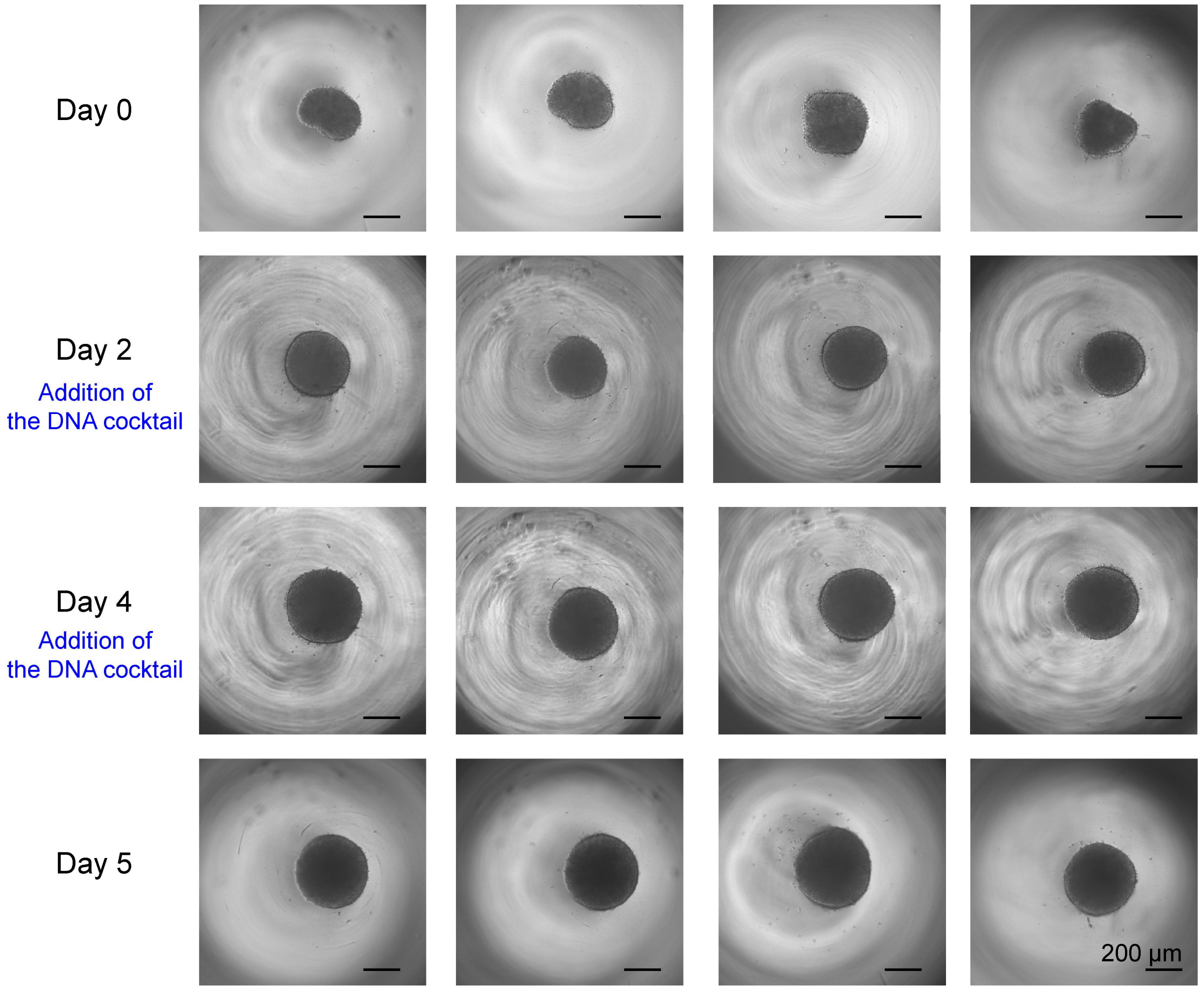

Figure S14. Bright-field microscopy images of the formation of four embryoid bodies for 5 days with the DNA cocktail added at day 2 and day 4 ([scaffold] = 1 nM; [staple] = 40 nM). All scale bars are 200 µm. Note that the imaging through the V-shaped bottom of the wells results in optically distorted images.

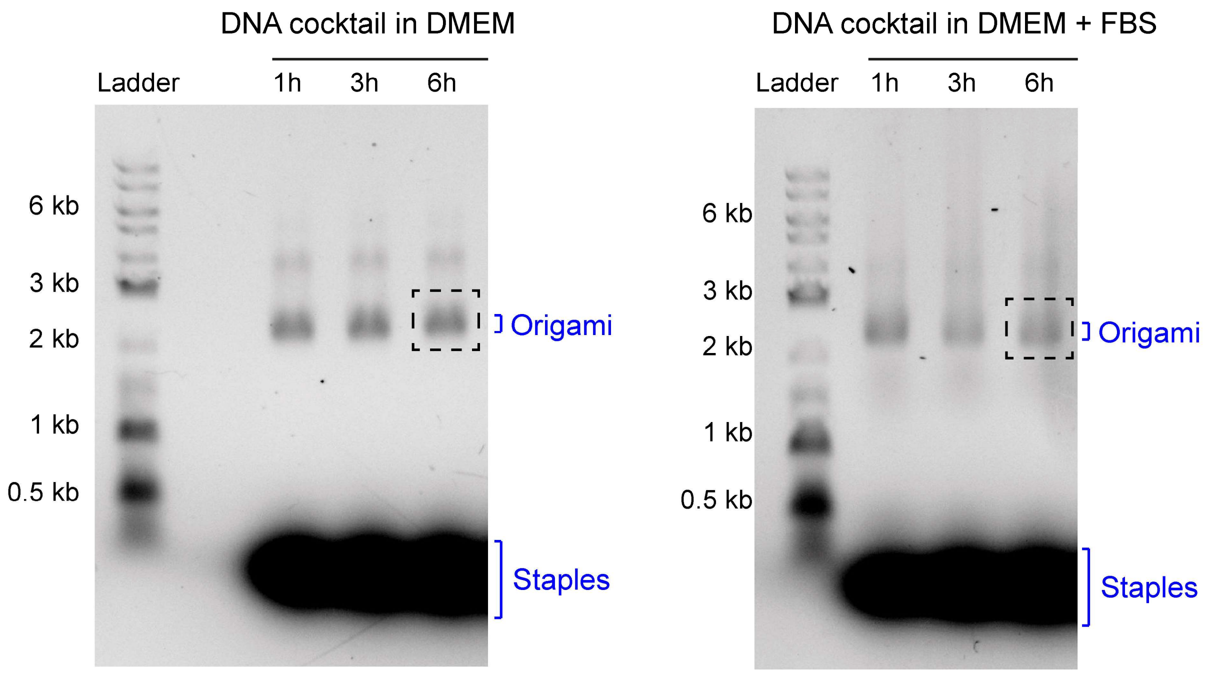

Figure S15. Electrophoresis agarose gel of the DNA cocktail coding for triangles in DMEM (left image) or DMEM supplemented with 10 vol% FBS (right image), incubated at 37°C for 1 h, 3 h, and 6 h. The dashed squares show the bands used for extraction and AFM observation in Figs. 1B and S1. [scaffold] = 10 nM; [staple] = 100 nM.

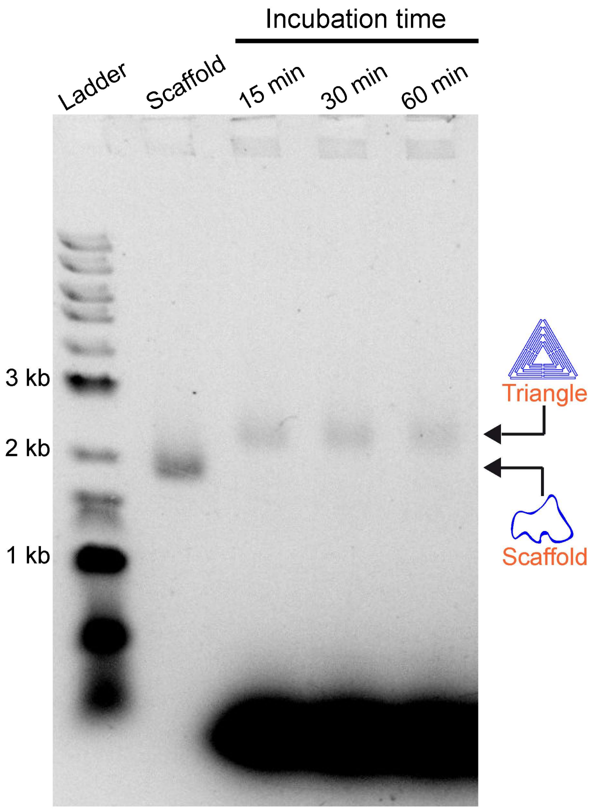

Figure S16. Electrophoresis agarose gel of HEK cell medium containing the DNA cocktail coding for triangle origami structures, as a function of incubation time with the cells in culture at 37°C. [scaffold] = 1 nM; [staple] = 40 nM.

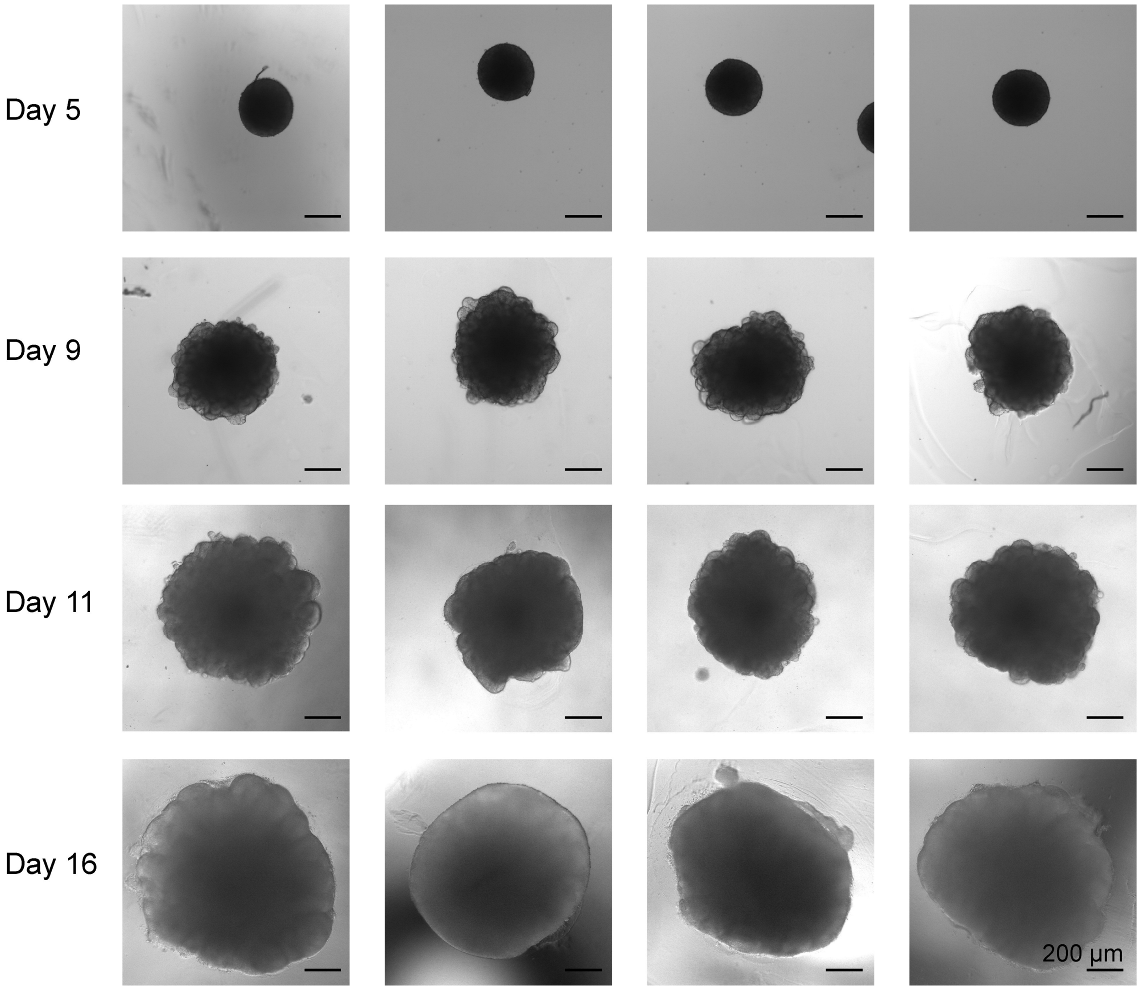

Figure S17. Bright-field microscopy images showing the evolution of four different embryoid bodies after *in situ* self-assembly of DNA origami structures (day 5) into cerebral organoids (day 16). All scale bars are 200 µm.

4) Supplementary tables

Table S1. Concentrations of the main monovalent and divalent cations present in common biological media as reported by Thermo Fisher technical resources (https://www.thermofisher.com/fr/fr/home/technical-resources/media-formulation.html as of 20 January 2025). The cation concentrations in Essential 8 was calculated from reported composition (3), including DMEM F12 (Thermo Fisher technical resources) and additional salts.

	DMEM HG	RPMI	Essential 8	PBS
**Main monovalent cations**	**$[Na^+]$ = 155 mM**<br>**$[K^+]$ = 5 mM**	**$[Na^+]$ = 133 mM**<br>**$[K^+]$ = 5 mM**	**$[Na^+]$ = 158 mM**<br>**$[K^+]$ = 4 mM**	**$[Na^+]$ = 158 mM**<br>**$[K^+]$ = 1 mM**
**Main divalent cations**	**$[Mg^{2+}]$ = 1 mM**<br>**$[Ca^{2+}]$ = 2 mM**	**$[Mg^{2+}]$ = 0.5 mM**<br>**$[Ca^{2+}]$ = 0.5 mM**	**$[Mg^{2+}]$ = 1 mM**<br>**$[Ca^{2+}]$ = 1 mM**	**$[Mg^{2+}]$ = 0**<br>**$[Ca^{2+}]$ = 0**

Table S2. Number n of analysed nanostructures and yield of perfectly folded DNA origami nanostructures ρ for each condition displayed in Fig. 1D.

**Incubation Time** / **[scaffold]; [staples]**	**5 min**	**15 min**	**30 min**	**60 min**
**1 nM; 10 nM**	No structures detected	n = 108	n = 190	n = 177
		ρ = 0 %	ρ = 7 %	ρ = 25 %
**1 nM; 40 nM**	n = 158	n = 165	n = 124	n = 204
	ρ = 0 %	ρ = 16 %	ρ = 27 %	ρ = 35 %
**10 nM; 400 nM**	n = 144	n = 116	n = 256	n = 515
	ρ = 41 %	ρ = 33 %	ρ = 42 %	ρ = 57 %

Table S3. Number *n* of analysed nanostructures and yield of perfectly folded DNA origami nanostructures *ρ* for each condition displayed in Fig. 3B.

Cell culture time \ Incubation Time		5 min	15 min	60 min
**HEK**	**2 days**	$n = 96$	$n = 238$	$n = 92$
		$\rho = 47$ %	$\rho = 33$ %	$\rho = 37$ %
	**3 days**	$n = 69$	$n = 82$	$n = 386$
		$\rho = 32$ %	$\rho = 32$ %	$\rho = 39$ %
**HeLa**	**2 days**	$n = 79$		
		$\rho = 37$ %		

5) Legend of the Supplementary Movie S1

Real-time fluorescence video of the self-assembled DNA nanotubes in DMEM at 37 °C overnight.

6) Supplementary References

1. P. W. K. Rothemund, Folding DNA to create nanoscale shapes and patterns. *Nature* **440**, 297–302 (2006).
2. L. N. Green, *et al.*, Autonomous dynamic control of DNA nanostructure self-assembly. *Nat Chem* **11**, 510–520 (2019).
3. G. Chen, *et al.*, Chemically defined conditions for human iPSC derivation and culture. *Nat Methods* **8**, 424–429 (2011).
